# Supplementary material for: Intersection of gambling with smoking and alcohol use in Great Britain: a cross-sectional survey in October 2022
Source: BMJ Open. 2024 Apr 10;14(4):e079633. doi: 10.1136/bmjopen-2023-079633 (PMC11015260; doi:10.1136/bmjopen-2023-079633)
Supplement: Supplementary data [file bmjopen-2023-079633supp002.pdf]

Supplementary appendix

Measures

Past-year gambling

Affirmative responses to any of the gambling types listed below were used to classify individuals as having gambled in the past year.

- National lottery, other lotteries, or scratch cards
- Football pools
- Bingo (not online)
- Slot machines
- Machines in a bookmakers
- Casino table games (not online)
- Online gambling in slots, casino, or bingo
- Online betting with a bookmaker
- Betting exchange
- Horse races (not online)
- Dog races (not online)
- Sports events (not online)
- Private betting
- Loot boxes or skins gambling within online/video games
- Crypto casinos
- Any other gambling event or activity

Problem gambling severity index (PGSI)

The PGSI is a nine item questionnaire on gambling severity and was derived from the Canadian Problem Gambling Index [22,23], and asked to those categorized as having gambled in the past year:

"Thinking about the last 12 months…"

1. Have you bet more than you could really afford to lose?
2. Have you needed to gamble with larger amounts of money to get the same feeling of excitement?
3. When you gambled, did you go back another day to try to win back the money you lost?
4. Have you borrowed money or sold anything to get money to gamble?
5. Have you felt that you might have a problem with gambling?
6. Has gambling caused you any health problems, including stress or anxiety?
7. Have people criticized your betting or told you that you had a gambling problem, regardless of whether or not you thought it was true?
8. Has your gambling caused any financial problems for you or your household?
9. Have you felt guilty about the way you gamble or what happens when you gamble?

For each item the respondent indicates one of the following: Never/None of the time (scored 0); Sometimes (1); Most of the time (2); Almost always (3). A sum score with a possible range from 0 to 27 is calculated. Scores between 1 and 7 represent "at risk" gambling (one to four "low risk" and five to seven "moderate risk"). An individual scoring eight or higher is classified as a

"disordered gambler" [24]. For this study, a category of "any-risk" will refer to those scoring 1 or greater.

Weekly expenditure on gambling, smoking and alcohol

A sum of weekly expenditure on gambling, smoking and alcohol was derived from responses to three questions on expenditure on each:

"On average about how much per week do you think you spend on [gambling/cigarettes or tobacco/alcohol for your own consumption]?"

Smoking status

Smoking status was ascertained using the following question and response options:

"Which of the following best applies to you?"

1. I smoke cigarettes (including hand rolled) every day
2. I smoke cigarettes (including hand rolled), but not every day
3. I do not smoke cigarettes at all, but I do smoke tobacco of some kind (e.g. Pipe, Cigar or Shisha)
4. I have stopped smoking completely in the last year
5. I stopped smoking completely more than a year ago
6. I have never been a smoker (ie. smoked for a year or more)

Responses of 1, 2 or 4 above were classified as past-year cigarette smokers, 4 or 5 as ex-smokers and 6 as never smokers.

Those who indicated that they do not smoke cigarettes but do smoke tobacco of some kind (answer 3 above) were excluded from the analysis because they did not respond to measures that assess dependence in cigarette smokers (cigarettes per day and time to first cigarette after waking).

Level of alcohol consumption (AUDIT-C score)

Heaviness of the last six months drinking was assessed using the consumption items from the Alcohol Use Disorders Identification Test (AUDIT-C) [26], a three-item screening tool developed by the World Health Organisation, with a score ranging from 0 to 12. Respondents scoring 4 or higher on the AUDIT-C are typically classified as drinking alcohol at increasing and high-risk. For sample characteristics, AUDIT-C was reported as a binary categorical variable (scores less than 4 vs 4 or greater) while for regression modelling it was included as a continuous variable.

Sociodemographic covariates

We used the National Readership Survey's classification social grade based on occupation (ABC1: higher and intermediate managerial, administrative, and professional, supervisory, clerical and junior managerial, administrative and professional; C2DE: skilled manual workers, semi-skilled and unskilled manual workers and state pensioners, casual and lowest-grade workers, unemployed with state benefits.)[25] .

Sociodemographic covariates included age (18-24, 25-34, 35-44, 45-54 and 65+) identified sex (Women, Men and 'In another way'/refused; all response options were reported in sample characteristics, but due to small case numbers of "in another way/refused" this category was excluded from regression analyses), and region of Great Britain (North, Midlands, South, Scotland and Wales).

Table S1: Types of gambling

Type of gambling	Overall (n=2398)	Smoking (n=345)	Audit-c >4 (n=775)
National lottery, other lotteries of scratch cards	38.4 % (850)	43.8% (138)	45.8% (330)
Football pools	2.1 % (45)	2.3% (8)	4.0% (29)
Online gambling in slots, casino or bingo	4.1% (92)	7.6% (24)	7.9% (57)
Bingo (not online)	3.4% (75)	5.7% (18)	4.8% (35)
Casino table games (not online)	1.7% (39)	2.6% (8)	3.5% (25)
Slot machines	2.8% (62)	6.3% (20)	5.9% (43)
Online betting with bookmaker	5.5% (122)	6.0% (19)	8.0% (58)
Machines in a bookmaker	0.4% (10)	0.0% (0)	0.9% (7)
Horse races (not online)	4.8% (106)	4.9% (16)	7.6% (55)
Dog races (not online)	0.5% (12)	0.3% (1)	0.4% (3)
Sports evens (not online)	1.9% (42)	2.2% (7)	2.8% (20)
Betting exchange	0.4% (9)	0.0% (0)	0.4% (3)
Private betting	0.5% (10)	0.0% (0)	0.6% (4)
Loot boxes or skins gambling within online video games	1.5% (33)	1.9% (6)	1.7% (13)
Crypto casinos	0.0%	0.0% (0)	0.0% (0)
Any other gambling activity	1.6% (36)	1.4% (4)	2.1% (15)

Percentages are weighted

Table S2: Full model output for association between current cigarette smoking, or drinking at increasing and higher risk levels, and any risk gambling according to the PGSI*

Variable	Event Rate	OR	95% CI	P
**Current cigarette smoking**				
**PGSI category**				
No risk	125 / 897 (14%)	—	—	
Any risk	10 / 57 (18%)	0.80	0.35, 1.66	0.57
**Age**				
18-24	22 / 82 (27%)	—	—	
25-34	36 / 142 (25%)	0.94	0.50, 1.82	0.86
35-44	16 / 146 (11%)	0.36	0.17, 0.76	0.007
45-54	23 / 191 (12%)	0.37	0.18, 0.73	0.004
55-64	24 / 204 (12%)	0.33	0.16, 0.64	0.001
65+	14 / 189 (7%)	0.18	0.08, 0.38	<0.001
Sex				

Men	70 / 499 (14%)	—	—	
Women	65 / 455 (14%)	1.01	0.68, 1.49	0.98
**Region**				
South	44 / 310 (14%)	—	—	
Midlands	24 / 217 (11%)	0.75	0.42, 1.29	0.30
North	36 / 229 (16%)	1.05	0.63, 1.73	0.86
Wales	9 / 69 (13%)	1.09	0.46, 2.36	0.83
Scotland	22 / 129 (17%)	1.20	0.66, 2.16	0.54
**Social grade**				
AB	15 / 241 (6%)	—	—	
C1	54 / 427 (13%)	2.06	1.15, 3.89	0.020
C2	30 / 157 (19%)	3.53	1.82, 7.07	<0.001
D	18 / 69 (26%)	5.15	2.37, 11.3	<0.001
E	18 / 60 (30%)	8.14	3.66, 18.4	<0.001
**Drinking at increasing and higher risk levels**				
**PGSI category**				
No risk	363 / 885 (41%)	—	—	
Any risk	26 / 58 (45%)	0.94	0.52, 1.69	0.83
**Age**				
18-24	48 / 80 (60%)	—	—	
25-34	54 / 140 (39%)	0.42	0.23, 0.76	0.004
35-44	72 / 146 (49%)	0.62	0.35, 1.11	0.11
45-54	75 / 190 (39%)	0.44	0.25, 0.76	0.004
55-64	90 / 202 (45%)	0.54	0.31, 0.94	0.029
65+	50 / 185 (27%)	0.26	0.14, 0.46	<0.001
**Sex**				
Men	247 / 491 (50%)	—	—	
Women	142 / 452 (31%)	0.45	0.34, 0.59	<0.001
**Region**				
South	115 / 304 (38%)	—	—	
Midlands	85 / 213 (40%)	1.14	0.78, 1.67	0.49
North	110 / 235 (47%)	1.63	1.13, 2.35	0.010
Wales	22 / 66 (33%)	0.89	0.49, 1.59	0.70
Scotland	57 / 125 (46%)	1.40	0.89, 2.19	0.14
**Social grade**				
AB	102 / 241 (42%)	—	—	
C1	182 / 421 (43%)	1.04	0.75, 1.46	0.81
C2	73 / 153 (48%)	1.14	0.74, 1.75	0.55
D	27 / 69 (39%)	0.83	0.46, 1.46	0.51

E	5 / 59 (9%)	0.13	0.04, 0.32	<0.001

Table S3: Bayes factors for a range of hypothetical effect sizes related to the association between any risk of harm (according to the PGSI) and smoking status or drinking at increasing and higher risk levels.

Observed OR (95% CI)	Hypothesized OR	Bayes Factor
**PGSI and smoking status**		
	0.50	0.54
	0.90	0.98
0.80 (0.35 - 1.66)	1.10	0.98
	1.50	0.75
	1.90	0.57
**PGSI and AUDIT-C score**		
	0.50	0.40
	0.90	0.95
0.94 (0.52 - 1.69)	1.10	0.95
	1.50	0.60
	1.90	0.43

www.bayesfactor.info

Table S4: Weekly expenditure (in £) on i) gambling according to PGSI, smoking and AUDIT-C scores and ii) expenditure on gambling, smoking and alcohol among people who smoked and/or scored 4 or greater on the AUDIT-C.

	Mean	SD	Lower CI	Upper CI	Median	Min	Max
**Weekly expenditure on gambling**							
**Overall** (n=945)	7.69	37.60	5.17	10.21	3.00	0.00	998.00
**PGSI**							
PGSI No risk (n=878)	4.80	7.68	4.18	5.43	2.00	0.00	75.00
PGSI Any risk (n=67)	45.68	134.13	12.07	79.29	3.00	0.00	998.0
**Smoking status**							
Not smoking (n=784)	7.61	39.67	4.69	10.53	3.00	0.00	998.00
Currently smoking (n=143)	8.09	26.69	3.52	12.65	3.00	0.00	300.00
**AUDIT-C score**							
<4 (n=539)	5.26	10.20	4.27	6.25	2.00	0.00	100.00
4 or higher (n=380)	10.74	56.02	4.86	16.66	3.00	0.00	998.00
**Weekly expenditure on gambling and smoking overall and according to PGSI**							
**Overall** (n=135)	42.73	42.40	33.88	51.59	29.40	2.00	301.00
**PGSI**							
PGSI No Risk (n=124)	38.71	34.48	30.73	46.68	28.00	2.00	170.00
PGSI Any Risk (n=11)	88.94	85.51	36.09	141.80	49.15	18.00	301.00
**Weekly expenditure on gambling and alcohol overall and according to PGSI**							
**Overall** (n=340)	36.48	87.17	26.83	46.13	24.00	1.00	1497.00
**PGSI**							
PGSI No Risk (n=313)	28.29	22.41	25.28	31.30	22.00	1.00	125.00
PGSI Any Risk (n=27)	131.42	288.88	21.81	241.04	50.80	3.00	1497.00
**Weekly expenditure on gambling, smoking and alcohol overall and according to PGSI**							

**Overall** (n=57)	69.37	58.67	48.78	89.96	51.00	3.00	270.00
**PGSI**							
PGSI No Risk (n=52)	66.83	59.75	44.94	88.72	48.92	3.00	270.00
PGSI Any Risk (n=5)	97.22	42.30	64.37	130.06	74.17	30.00	126.00

Figure S1: Box plots for the distribution of mean weekly expenditure (log transformed scale) on gambling according to no risk, or any risk of harm from gambling (scores >0 on the PGSI)

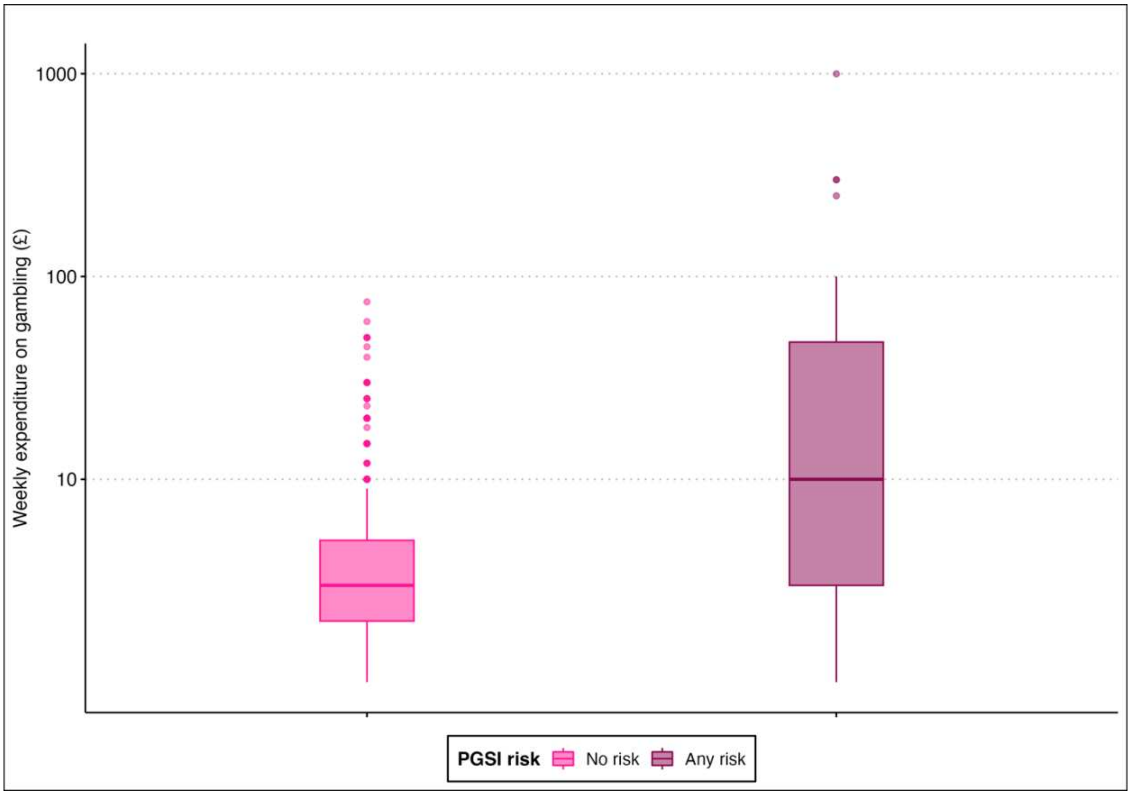

The distribution of mean weekly spend on gambling highlights how the mean is influenced by a small number of higher values in the any risk category:
