# Supplementary material for: Intersection of gambling with smoking and alcohol use in Great Britain: a cross-sectional survey in October 2022
Source: BMJ Open. 2024 Apr 10;14(4):e079633. doi: 10.1136/bmjopen-2023-079633 (PMC11015260; doi:10.1136/bmjopen-2023-079633)
Supplement: Author's manuscript [file bmjopen-2023-079633.draft_revisions.pdf]

BMJ Open is committed to open peer review. As part of this commitment we make the peer review history of every article we publish publicly available.

When an article is published we post the peer reviewers' comments and the authors' responses online. We also post the versions of the paper that were used during peer review. These are the versions that the peer review comments apply to.

The versions of the paper that follow are the versions that were submitted during the peer review process. They are not the versions of record or the final published versions. They should not be cited or distributed as the published version of this manuscript.

BMJ Open is an open access journal and the full, final, typeset and author-corrected version of record of the manuscript is available on our site with no access controls, subscription charges or pay-per-view fees (http://bmjopen.bmj.com).

If you have any questions on BMJ Open's open peer review process please email info.bmjopen@bmj.com

BMJ Open

The Intersection of Gambling with Smoking and Alcohol use in Great Britain: A Cross-Sectional Survey in October 2022

Journal:	*BMJ Open*
Manuscript ID	bmjopen-2023-079633
Article Type:	Original research
Date Submitted by the Author:	06-Sep-2023
Complete List of Authors:	Kock, Loren; University College London Research Department of Epidemiology and Public Health, Behavioural Science and Health; Dept. Behavioural Science & Health<br>Cox, Sharon; University College London, Behavioural Science and Health; SPECTRUM Research Consortium,<br>Shahab, Lion; UCL, Dept of Epidemiology and Public Health<br>Roberts, Amanda; University of Lincoln<br>Sharman, Steve; King's College London,<br>Buss, Vera; University College London<br>Brown, Jamie; University College London, Psychology & Language Sciences
Keywords:	Behavior, EPIDEMIOLOGY, Substance misuse < PSYCHIATRY

SCHOLARONE™
Manuscripts

I, the Submitting Author has the right to grant and does grant on behalf of all authors of the Work (as defined in the below author licence), an exclusive licence and/or a non-exclusive licence for contributions from authors who are: i) UK Crown employees; ii) where BMJ has agreed a CC-BY licence shall apply, and/or iii) in accordance with the terms applicable for US Federal Government officers or employees acting as part of their official duties; on a worldwide, perpetual, irrevocable, royalty-free basis to BMJ Publishing Group Ltd ("BMJ") its licensees and where the relevant Journal is co-owned by BMJ to the co-owners of the Journal, to publish the Work in this journal and any other BMJ products and to exploit all rights, as set out in our licence.

The Submitting Author accepts and understands that any supply made under these terms is made by BMJ to the Submitting Author unless you are acting as an employee on behalf of your employer or a postgraduate student of an affiliated institution which is paying any applicable article publishing charge ("APC") for Open Access articles. Where the Submitting Author wishes to make the Work available on an Open Access basis (and intends to pay the relevant APC), the terms of reuse of such Open Access shall be governed by a Creative Commons licence – details of these licences and which Creative Commons licence will apply to this Work are set out in our licence referred to above.

Other than as permitted in any relevant BMJ Author's Self Archiving Policies, I confirm this Work has not been accepted for publication elsewhere, is not being considered for publication elsewhere and does not duplicate material already published. I confirm all authors consent to publication of this Work and authorise the granting of this licence.

For peer review only

The Intersection of Gambling with Smoking and Alcohol use in Great Britain: A Cross-Sectional Survey in October 2022

Loren Kock[1,2], Sharon Cox[1,2], Lion Shahab[1,2], Amanda Roberts[3], Steve Sharman[4] Vera Buss[1,2], Jamie Brown[1,2]

[1]Department of Behavioural Science and Health, University College London, UK
[2]SPECTRUM Research Consortium, Edinburgh, UK
[3]School of Psychology, University of Lincoln, UK
[4]National Addiction Centre, King's College London, UK

Correspondence to: Dr Loren Kock (l.kock@ucl.ac.uk)

Word count: 3,031

For peer review only

ABSTRACT

Objectives: Gambling is often associated with cigarette smoking and alcohol consumption. This study aimed to explore the intersection of gambling across all risk levels harm with smoking and alcohol use among adults in Great Britain.

Design: A nationally representative cross-sectional survey in October 2022.

Setting: Great Britain.

Participants: A weighted total of 2,398 adults (18+ years).

Outcome measures: We examined the prevalence of gambling in the past year and, among those reporting gambling, assessed the associations between any risk of harm from gambling (scores of >0 on the problem gambling severity index) and cigarette smoking and higher risk alcohol use. We also explored the average weekly expenditure on gambling, smoking and alcohol use among those categorised at any-risk of harm from gambling.

Results: Overall, 43.6% (95% CI 41.2-45.9) of adults gambled in the past year. Among those who gambled in the past year 7.3% (5.3-9.3) were classified at any-risk of harm from gambling, 16.0% 13.2-18.8) were currently smoking, and 40.8% (37.2-44.4) were drinking at increasing and higher risk levels. There were no apparent associations between any risk of harm from gambling and current cigarette smoking (ORadj=0.80, 95% CI 0.35-1.66) or drinking at increasing and higher risk levels (ORadj=0.94, 0.52-1.69), respectively. Analyses using Bayes factors indicated that these data were insensitive to distinguish no effect from a range of associations (OR=0.5-1.9). The mean weekly spend on gambling was £7.69 (95% CI 5.17-10.21) overall, and £45.68 (12.07-79.29) among those at any risk of harm from gambling.

Conclusions: Pilot data in a population-level survey on smoking and alcohol use yielded estimates of gambling participation and at-risk gambling that are similar to other population-level surveys. Further data are needed to elucidate the intersections more reliably between gambling, smoking and alcohol use, and inform population-level approaches to reduce harms conferred by these behaviors.

Keywords: Disordered gambling, smoking, alcohol use

For peer review only - http://bmjopen.bmj.com/site/about/guidelines.xhtml

Strength and limitations

- This study benefitted from the use of the Smoking and Alcohol Toolkit Study, a representative survey in Great Britain that includes detailed measurement of smoking and alcohol use.
- The study assessed the association between any-risk of harm from gambling and cigarette smoking or increasing and high risk alcohol use.
- The study also explored weekly expenditure on gambling, smoking and alcohol use among those categorised at any-risk of harm from gambling.
- The limitations of this study include the data being cross-sectional and self-reported and that a small number of respondents were classified as at any-risk of harm form gambling due to data being collected in one month.

For peer review only

For peer review only - http://bmjopen.bmj.com/site/about/guidelines.xhtml

INTRODUCTION

General population surveys estimate that half of adults in Great Britain have gambled in the previous 12 months [1,2]. Although many individuals gamble as a social activity without experiencing harm, some gamble at levels that adversely impact financial stability, personal and family wellbeing and physical and mental health [3,4]. Conservative estimates indicate that approximately 0.3-0.5% of the general UK adult population report severe gambling behaviours that warrant a diagnosis of gambling disorder (hereafter termed "disordered gambling") and 3-4% are "at-risk" (those who experience a low or moderate level of problems leading to some negative consequences) [2,5]. Due to a combination of financial and health costs associated with gambling (including homelessness, suicide, depression, alcohol dependence, illicit substance use, unemployment and imprisonment), gambling is also associated with an estimated annual economic cost to the UK government of ~£413 million, and potentially £655-£1,355 million in societal value due to adverse health effects [6].

Gambling is thought to be associated with other addictive behaviours, including cigarette smoking and excess substance use such as alcohol consumption [7,8], and this may relate to common neurobiological, genetic and social/environmental factors which could act to reinforce each respective behaviour [9–11]. Previous prevalence surveys in Great Britain have illustrated nuance in the relationship between disordered gambling and use of these substances. The 2007 British Gambling survey indicated that smoking was associated with higher rates of past-year gambling (79% vs 64% in those who did not smoke) and disordered gambling (1.4% vs 0.4%), while the prevalence of disordered gambling was 3.4% in those who consumed >20 units of alcohol on their heaviest drinking day and 0.1% who consumed 1-4 units. [12]. In contrast, a 2021 evidence review on gambling related harms conducted by Public Health England concluded that although increased alcohol consumption was associated with gambling at all levels of harm, there was no apparent association with smoking [2].

The co-occurrence with smoking and increasing and higher risk alcohol consumption is likely to compound the physical, social, financial and psychological harms that each of them cause. These harms may be disproportionately greater for certain sub-groups, namely those experiencing poverty [13] who are also more likely to smoke and report greater harms from alcohol consumption compared with more advantaged groups [14,15]. Expenditure on all three may be particularly concerning for individuals and families on lower incomes and those experiencing financial difficulty, which is increasingly common following the COVID-19 pandemic and global inflation in 2022. In the UK and elsewhere, rising prices for everyday items and services have resulted in less 'disposable' income, termed the 'cost-of-living' crisis, which has had a worsening impact on more lower income households [16].

To build on the existing research reporting on these three behaviours, we piloted the addition of several standard gambling measures to an ongoing representative monthly survey of smoking and alcohol use in Great Britain. The objective of this study is to explore the intersection of gambling across all risk levels (henceforth termed "any-risk" gambling) with smoking and alcohol use among adults in Great Britain. Specifically, we aimed to i) estimate the prevalence of past-year gambling according to smoking and increasing and higher risk alcohol use, ii) assess the associations between any-risk gambling (defined by scores of >0 on the problem gambling severity index) and smoking and increasing and higher risk alcohol use, respectively and iii) explore the average weekly expenditure on gambling, smoking and alcohol use among those reporting any-risk gambling.

For peer review only - http://bmjopen.bmj.com/site/about/guidelines.xhtml

METHODS

Sample and recruitment

The study population consisted of adults aged ≥18 and over living in households in Great Britain, surveyed in October 2022 in the Smoking and Alcohol Toolkit Study (STS/ATS). Ethical approval for the STS/ATS was granted by the UCL Ethics Committee (ID 0498/001). The data were not collected by UCL and were anonymised when received by the research team.

The STS/ATS uses a hybrid of random location and quota sampling to select a new sample of approximately 2,400 adults (aged ≥16 years) each month in Great Britain. Telephone interviews are carried out with one household member until quotas based on factors influencing the probability of being at home (e.g., gender, age, working status) are fulfilled. We used survey weighting to match descriptive data to sociodemographic profile in Great Britain (based on age, social grade, region, tenure, ethnicity and working status within sex). Detailed survey methodology is reported elsewhere [17,18]. Comparisons with sales data and other national surveys show that the STS recruits a representative sample of the population in Great Britain with regard to key demographic variables and smoking indicators.

For the current study, all adults were asked a question pertaining to past year gambling participation (derived from indications of type of gambling). Due to funding constraints, questions used to derive the Problem Gambling Severity Index and weekly expenditure on gambling were asked to a partial sample consisting of ~88% of the total eligible sample of people who indicated that they gambled in the past year.

Measures

Past-year gambling

Affirmative responses to any of the gambling types in the question below were used to classify individuals as having gambled in the past year.

"From the list below please select up to 5 activities that you have participated in most frequently in the past year:"

- National lottery, other lotteries, or scratch cards
- Football pools
- Bingo (not online)
- Slot machines
- Machines in a bookmakers
- Casino table games (not online)
- Online gambling in slots, casino, or bingo
- Online betting with a bookmaker
- Betting exchange
- Horse races (not online)
- Dog races (not online)
- Sports events (not online)
- Private betting
- Loot boxes or skins gambling within online/video games
- Crypto casinos
- Any other gambling event or activity

For peer review only - http://bmjopen.bmj.com/site/about/guidelines.xhtml

Problem gambling severity index (PGSI)

The PGSI is a nine item questionnaire on gambling severity and was derived from the Canadian Problem Gambling Index [19,20], and asked to those categorized as having gambled in the past year:

"Thinking about the last 12 months…"

1. Have you bet more than you could really afford to lose?
2. Have you needed to gamble with larger amounts of money to get the same feeling of excitement?
3. When you gambled, did you go back another day to try to win back the money you lost?
4. Have you borrowed money or sold anything to get money to gamble?
5. Have you felt that you might have a problem with gambling?
6. Has gambling caused you any health problems, including stress or anxiety?
7. Have people criticized your betting or told you that you had a gambling problem, regardless of whether or not you thought it was true?
8. Has your gambling caused any financial problems for you or your household?
9. Have you felt guilty about the way you gamble or what happens when you gamble?

For each item the respondent indicates one of the following: Never/None of the time (scored 0); Sometimes (1); Most of the time (2); Almost always (3). A sum score with a possible range from 0 to 27 is calculated. Scores between 1 and 7 represent "at risk" gambling (one to four "low risk" and five to seven "moderate risk"). An individual scoring eight or higher is classified as a "disordered gambler". [21]. For this study, a category of "any-risk" will refer to those scoring 1 or greater.

Weekly expenditure on gambling, smoking and alcohol

A sum of weekly expenditure on gambling, smoking and alcohol was derived from responses to three questions on expenditure on each:

"On average about how much per week do you think you spend on [gambling/cigarettes or tobacco/alcohol for your own consumption]?"

Smoking status

Smoking status was ascertained using the following question and response options:

"Which of the following best applies to you?"

1. I smoke cigarettes (including hand rolled) every day
2. I smoke cigarettes (including hand rolled), but not every day
3. I do not smoke cigarettes at all, but I do smoke tobacco of some kind (e.g. Pipe, Cigar or Shisha)
4. I have stopped smoking completely in the last year
5. I stopped smoking completely more than a year ago
6. I have never been a smoker (ie. smoked for a year or more)

Responses of 1, 2 or 4 above were classified as past-year cigarette smokers, 4 or 5 as ex-smokers and 6 as never smokers.

For peer review only - http://bmjopen.bmj.com/site/about/guidelines.xhtml

Those who indicated that they do not smoke cigarettes but do smoke tobacco of some kind (answer 3 above) were excluded from the analysis because they did not respond to measures that assess dependence in cigarette smokers (cigarettes per day and time to first cigarette after waking).

Level of alcohol consumption (AUDIT-C score)

Heaviness of the last six months drinking was assessed using the consumption items from the Alcohol Use Disorders Identification Test (AUDIT-C) [22], a three-item screening tool developed by the World Health Organisation, with a score ranging from 0 to 12. Respondents scoring 4 or higher on the AUDIT-C are typically classified as drinking alcohol at increasing and high-risk. For sample characteristics, AUDIT-C was reported as a binary categorical variable (scores less than 4 vs 4 or greater) while for regression modelling it was included as a continuous variable.

Sociodemographic covariates

We used the National Readership Survey's classification social grade based on occupation (ABC1: higher and intermediate managerial, administrative, and professional, supervisory, clerical and junior managerial, administrative and professional; C2DE: skilled manual workers, semi-skilled and unskilled manual workers and state pensioners, casual and lowest-grade workers, unemployed with state benefits.)[23]

Sociodemographic covariates included age (18-24, 25-34, 35-44, 45-54 and 65+) identified sex (Women, Men and 'In another way'/refused; all response options were reported in sample characteristics, but due to small case numbers of "in another way/refused" this category was excluded from regression analyses), and region of Great Britain (North, Midlands, South, Scotland and Wales).

Analyses

The analyses were pre-registered on the open science framework, https://osf.io/nc6jm and conducted in R version 4.2.2 (packages *tidyverse* and *survey* [24,25]) with all statistical code made open-access at https://osf.io/aj7c9/. The study followed the Strengthening the Reporting of Observational Studies in Epidemiology (STROBE) guidelines for cross-sectional studies.

Characteristics of the sample and descriptive statistics are presented using weighted descriptive statistics. The prevalence of past-year gambling and any-risk gambling (according to the PGSI) are presented weighted with 95% confidence intervals. Estimates are reported both as a percentage of the overall population, and of those who gambled in the past-year (in the case of any-risk gambling).

Respondents with missing data on any of the covariates of interest (3.4% of total sample) are excluded from the analyses. We constructed logistic regression models to assess the associations between any-risk gambling with current smoking, increasing and higher risk alcohol consumption, respectively among people who gambled in the past year. All models are adjusted for key sociodemographic characteristics (age, sex, social grade, and region).

We estimated the average weekly expenditure on i) gambling and ii) gambling, smoking and alcohol use, among those classified as "any-risk" compared with those who gamble without risk. These data are presented descriptively as mean expenditure with measures of spread (standard error, 95% CI and range).

For peer review only - http://bmjopen.bmj.com/site/about/guidelines.xhtml

Unregistered changes to analysis plan

Observed non-significant associations between any risk of gambling harm and current smoking, or drinking at increasing and higher risk levels could have indicated evidence for no association, or that the data were insensitive to detect an effect. To explore this, post-hoc Bayes factors are calculated for a range of hypothetical effect sizes including the potential for lower odds (OR=0.5 or 0.9) or higher odds (OR=1.1, 1.5, or 1.9) of any risk of gambling harm according to cigarette smoking and AUDIT-C scores of four or more, respectively. Bayes factors were computed using an online calculator (www.bayesfactor.info).

RESULTS

A weighted total of 2,398 adults aged 18 and older (mean [SE] age = 47.7 [0.46]) surveyed in October 2022 were included in the analytic sample (Table 1). In the overall sample, 43.6% (95% CI 41.2-45.9) of adults participated in a gambling activity in the past year, and 3.2% (2.3-4.1) were classified as having any-risk of harm from gambling (i.e., scoring >0 on the PGSI), 14.5% (12.8-16.3) were currently smoking, and 33.4% (31.2-35.7) were drinking at increasing and higher risk levels. Among those who reported any gambling activity in the past year (n=1,045), 7.3% (5.3-9.3) were classified as being at any-risk of harm from gambling with 0.3% (0.0-0.66) classified as disordered gambling, 16.0% (13.2-18.8) were currently smoking, and 40.8% (37.2-44.4) were drinking at increasing and higher risk levels (Table 1). Aside from gambling on the national lottery, other lotteries or scratch cards (38.4%), the three most common gambling activities overall were online betting with bookmaker (5.5%), horse races (not online) (4.8%) and online gambling in slots, casino or bingo (4.1%) (Table S1).

There were no apparent associations between any risk of harm from gambling and current cigarette smoking (ORadj=0.80, 95% CI 0.35-1.66) or drinking at increasing and higher risk levels (ORadj=0.94, 95% CI 0.52-1.69), respectively (Table 2 and Table S2). Analyses using Bayes factors indicated that the data were insensitive to detect an effect in either direction and therefore these results are inconclusive (Table 3).

In the sample of adults who gambled in the past year, the mean weekly spend on gambling was £7.69 (95% CI 5.17-10.21) overall, and £45.68 (12.07-79.29) among those classified as at any risk of harm from gambling according to the PGSI (Table 4). The equivalent expenditure in a sample excluding those who only gambled on lottery/scratch cards was £13.45 (7.27-19.63) overall and £56.48 (14.79-98.17) in those at any risk of harm from gambling. Caution should be taken in the interpretation of expenditure in the any risk category due to a relatively small number of cases (n=67) compared with the no risk category (n=878). One respondent reported a weekly mean spend on gambling of £998.00. The distribution of mean weekly spend on gambling is shown in Figure 1 and highlights how the mean is influenced by a small number of higher values in the any risk category. According to smoking and drinking behavior, the mean weekly spend was £8.09 (3.52-12.65) in people currently smoking (vs. £7.61 in those not smoking) and £10.74 (4.86-16.66) among people drinking at increasing and higher risk levels (vs. £5.26 in people with AUDIT-C scores of< 4), respectively. The total expenditure on smoking, drinking and at-risk gambling among this intersection of people was £69.37 (49.78-89.96).

For peer review only

For peer review only - http://bmjopen.bmj.com/site/about/guidelines.xhtml

Table 1: Characteristics of sample

Characteristic	Overall n = 2,398	Did not gamble n = 1,353	Gambled in past year n = 1,045
**Age**			
18-24	13.7%, (328)	16.2%, (219)	10.5%, (110)
25-34	16.7%, (400)	16.3%, (221)	17.2%, (180)
35-44	15.4%, (370)	15.0%, (203)	16.0%, (168)
45-54	16.5%, (395)	14.8%, (201)	18.6%, (194)
55-64	15.0%, (359)	12.1%, (164)	18.6%, (194)
65+	22.7%, (544)	25.5%, (345)	19.1%, (199)
Missing	2	2	0
**Sex**			
Men	48.7%, (1,157)	46.5%, (622)	51.5%, (535)
Women	50.8%, (1,209)	53.1%, (710)	47.9%, (499)
In another way	0.5%, (12)	0.4%, (6)	0.6%, (6)
Missing	20	15	5
**Social grade**			
AB	26.1%, (626)	26.4%, (357)	25.8%, (269)
C1	29.9%, (716)	29.5%, (399)	30.3%, (316)
C2	20.3%, (486)	19.9%, (270)	20.7%, (216)
D	14.5%, (348)	14.2%, (193)	14.8%, (156)
E	9.3%, (222)	9.9%, (134)	8.4%, (88)
**Region**			
South	36.6%, (878)	36.1%, (489.2)	37.2%, (388.3)
Midlands	25.9%, (622)	25.5%, (345.7)	26.5%, (276.5)
North	23.8%, (571)	20.6%, (279.3)	27.9%, (291.6)
Wales	4.9%, (118)	6.2%, (84.0)	3.3%, (34.1)
Scotland	8.7%, (209)	11.5%, (155.2)	5.2%, (54.1)
**PGSI category**			
Did not gamble	56.4%, (1,353)	100.0%, (1,353)	-
No risk	40.4%, (969)	-	92.7%, (968.5)
Low risk	2.6%, (62)	-	5.9%, (62)
Moderate risk	0.5%, (11)	-	1.0%, (11)
Disordered gambling	0.1%, (3)	-	0.3%, (3)
**Smoked cigarettes**	14.5%, (345)	13.4%, (181)	16.0%, (164)
Missing	26	8	18
**AUDIT-C 4 or higher**			
4 or higher	33.4%, (775)	27.7%, (359)	40.8%, (415)
Missing	82	55	27

For peer review only - http://bmjopen.bmj.com/site/about/guidelines.xhtml

Table 2: Association between current cigarette smoking, or drinking at increasing and higher risk levels, and any risk gambling according to the PGSI*

Variable	Event Rate	OR	95% CI	P
**Current cigarette smoking**				
**PGSI category**				
No risk	125 / 897 (14%)	—	—	
Any risk	10 / 57 (18%)	0.80	0.35, 1.66	0.57
**Drinking at increasing and higher risk levels**				
**PGSI category**				
No risk	363 / 885 (41%)	—	—	
Any risk	26 / 58 (45%)	0.94	0.52, 1.69	0.83

Model adjusted for age, sex, social grade and region.

Table 3: Bayes factors for a range of hypothetical effect sizes related to the association between any risk of harm (according to the PGSI) and smoking status or drinking at increasing and higher risk levels.

Observed OR (95% CI)	Hypothesized OR	Bayes Factor
**PGSI and smoking status**		
	0.50	0.54
	0.90	0.98
0.80 (0.35 - 1.66)	1.10	0.98
	1.50	0.75
	1.90	0.57
**PGSI and AUDIT-C score**		
	0.50	0.40
	0.90	0.95
0.94 (0.52 - 1.69)	1.10	0.95
	1.50	0.60
	1.90	0.43

www.bayesfactor.info

Figure 1: The distribution of mean weekly expenditure (log transformed scale) on gambling according to no risk, or any risk of harm from gambling (scores >0 on the PGSI)

For peer review only - http://bmjopen.bmj.com/site/about/guidelines.xhtml

Table 4: Weekly expenditure (in £) on i) gambling according to PGSI, smoking and AUDIT-C scores and ii) expenditure on gambling, smoking and alcohol among people who reported data on smoking and drinking expenditure.

	Mean	SD	Lower CI	Upper CI	Median	Min	Max
**Weekly expenditure on gambling**							
**Overall** (n=945)	7.69	37.60	5.17	10.21	3.00	0.00	998.00
**PGSI**							
PGSI No risk (n=878)	4.80	7.68	4.18	5.43	2.00	0.00	75.00
PGSI Any risk (n=67)	45.68	134.13	12.07	79.29	3.00	0.00	998.0
**Smoking status**							
Not smoking (n=784)	7.61	39.67	4.69	10.53	3.00	0.00	998.00
Currently smoking (n=143)	8.09	26.69	3.52	12.65	3.00	0.00	300.00
**AUDIT-C score**							
<4 (n=539)	5.26	10.20	4.27	6.25	2.00	0.00	100.00
4 or higher (n=380)	10.74	56.02	4.86	16.66	3.00	0.00	998.00
**Weekly expenditure on gambling, smoking and alcohol, overall and according to PGSI**							
**Overall** (n=57)	69.37	58.67	48.78	89.96	51.00	3.00	270.00
**PGSI**							
PGSI No Risk (n=5)	66.83	59.75	44.94	88.72	48.92	3.00	270.00
PGSI Any Risk (n=52)	97.22	42.30	64.37	130.06	74.17	30.00	126.00

DISCUSSION

In a nationally representative survey of smoking and alcohol use in Great Britain, our pilot gambling questions collected during one month of data collection returned similar estimates for overall gambling participation, and for at-risk and disordered gambling as other national population surveys [1,6]. Approximately half of adults reported some gambling activity in the past year, and descriptively the prevalence of smoking (16%) and increasing and higher risk drinking (41%) was higher in those who gambled compared with those who did not (13% and 28%, respectively).

One in 14 people who gambled were classified as being at any risk of harm from gambling, but our data were likely insensitive to detect associations between any-risk of harm from gambling, smoking and higher risk drinking, if true associations ranging from OR=0.5 to 1.9 existed. Although gambling at any level of harm is consistently associated with alcohol consumption [2,26], the aetiology of this relationship is likely multidimensional. Observed associations in the wider literature may reflect common underlying genetic, social and environmental determinants [27,28], but also involve bi-directional causality whereby frequency of gambling is higher under the influence of alcohol [27]. Alcohol may be used as an avoidant coping mechanism following losses [29] and as a cued response following wins [30]. The mechanism through which alcohol consumption might lead to disordered gambling requires more research. For example, a recent review examining the salient hypothesis that acute alcohol consumption leads to harm from gambling by encouraging greater risk taking concluded that there was no reliable effect [31].

In 2009 a review into the comorbidity of smoking and gambling concluded that comorbidity was highly prevalent [32]. However, an evidence review published in 2023 conducted by Public Health England concluded that cigarette smoking was not associated with gambling among adults [2].

For peer review only - http://bmjopen.bmj.com/site/about/guidelines.xhtml

While this may not hold true in certain priority subgroups, further data examining this issue in the STS/ATS could provide important information at the population-level.

The paucity of data in our survey on individuals experiencing disordered gambling reflects the relatively small proportion of the population falling into this category, but also that population surveys cannot comprehensively capture relatively rare behaviours - like disordered gambling [33], or injecting drug use [34] – which are more common in subgroups of the population who fall outside of traditional sampling frames [35]. Nonetheless, because they are more numerous, the majority of harms from gambling at the population-level is driven by those classified as low, and moderate risk of harm [36]. Understanding the relationships between gambling behavior and other licit and commercially influenced addictive behaviors like smoking and alcohol use, and gambling at all levels of risk remains an important endeavour. While pilot data were collected in one survey month, extending data collection in a longer monthly time-series would allow these intersections to be interrogated with greater accuracy and reliability.

Finally, while there was wide uncertainty in our estimates for expenditure on gambling among those at any risk of harm from gambling, the outliers driving this uncertainty remain plausible given the extreme spending that can occur in those experiencing disordered gambling [36]. Indeed, due to the rise in online gambling in recent years, in their recent white paper the UK government has proposed introducing financial risk-checks for moderate to high spending [37]. While our estimates should be interpreted with caution, there was a signal for higher expenditure on gambling among those catagorised as drinking at an increasing and higher risk level. If true, this pattern of spending would conform to studies highlighting a positive relationship between increasing alcohol consumption and gambling spend [7].

In conclusion, the collection of pilot data on gambling in a population-level survey on smoking and alcohol use yielded estimates of gambling participation and at-risk and disordered gambling that are similar to other population-level surveys. Further data collection would help elucidate the intersections more reliably between gambling, smoking and alcohol use, and inform population-level approaches to reduce the harms to public health conferred by these behaviors.

Funding

Funding for STS/ATS data collection is supported by Cancer Research UK (PRCRPG-Nov21\100002), and the UK Prevention Research Partnership (MR/S037519/1).

Competing interest statement

JB reports receiving grants from Cancer Research UK during the conduct of the study and receiving unrestricted research funding from pharmaceutical companies who manufacture smoking cessation medications to study smoking cessation outside the submitted work. LS reports receiving honoraria for talks, receiving an unrestricted research grant and travel expenses to attend meetings and workshops by pharmaceutical companies that make smoking cessation products (Pfizer and Johnson & Johnson), and acting as a paid reviewer for grant-awarding bodies and as a paid consultant for health care companies. SC has provided expert consultancy to providers of UK life insurance and the pharmaceutical industry on matters relating to smoking cessation aids. SS has received funding from the Society for the Study of Addiction (SSA), and the NIHR. He is currently employed at the National Addictions Centre, part of the NIHR Biomedical Research Centre and declares no conflicts. AR has received funding from Santander, Public Health for Lincoln, The Royal Society, The Maurice and Jacqueline Bennett Charitable Trust, East

For peer review only - http://bmjopen.bmj.com/site/about/guidelines.xhtml

Midlands RDS and internal University of Lincoln awards and declares no conflicts of interest. LK, VB and EP have no conflicts of interests to declare.

Contributors: Dr. Kock had full access to all of the data in the study and takes responsibility for the integrity of the data and the accuracy of the data analysis.

Concept and design: Kock, Brown, Sharman, Roberts, Shahab, Cox
Acquisition, analysis, or interpretation of data: Kock
Drafting of the manuscript: Kock
Critical revision of the manuscript for important intellectual content: All authors
Statistical analysis: Kock
Obtained funding: Kock, Brown
Supervision: Brown

Data sharing statement

The analyses were pre-registered on the open science framework, https://osf.io/nc6jm and conducted in R version 4.2.2 (packages *tidyverse* and *survey* [24,25]) with all statistical code made open-access at https://osf.io/aj7c9/. Data are available from authors upon request.

REFERENCES

1. Conolly A, Davies B, Fuller E, Heinze N, Wardle H. Gambling behaviour in Great Britain in 2016 Evidence from England, Scotland and Wales [Internet]. 2018 [cited 2023 Jan 2]. Available from: https://assets.ctfassets.net/j16ev64qyf6l/60qlzeoSZIJ2QxByMAGJqz/e3af209d552b08c16566a217ed422e68/Gambling-behaviour-in-Great-Britain-2016.pdf
2. Public Health England. Gambling-related harms evidence review: summary [Internet]. 2021 [cited 2023 Jan 3]. Available from: https://www.gov.uk/government/publications/gambling-related-harms-evidence-review/gambling-related-harms-evidence-review-summary
3. Hodgins DC, Stea JN, Grant JE. Gambling disorders. The Lancet. 2011 Nov 26;378(9806):1874–84.
4. Petry NM, Zajac K, Ginley MK. Behavioral Addictions as Mental Disorders: To Be or Not To Be? Annu Rev Clin Psychol [Internet]. 2018 May 5 [cited 2023 Feb 2];14:399. Available from: /pmc/articles/PMC5992581/
5. Gambling Commission. Statistics on participation and problem gambling for the year to March 2023 [Internet]. 2023 [cited 2023 Jun 29]. Available from: https://www.gamblingcommission.gov.uk/statistics-and-research/publication/statistics-on-participation-and-problem-gambling-for-the-year-to-march-2023
6. Office for Health Improvement and Disparities. The economic cost of gambling-related harm in England: evidence update 2023. 2023.
7. Díaz A, Pérez L. Gambling and substance use: A cross-consumption analysis of tobacco smoking, alcohol drinking and gambling. https://doi.org/101080/0889707720211903657 [Internet]. 2021 [cited 2023 Jan 10];42(4):1016–21. Available from: https://cogentoa.tandfonline.com/doi/abs/10.1080/08897077.2021.1903657
8. Sharman S, Murphy R, Turner JJD, Roberts A. Trends and patterns in UK treatment seeking gamblers: 2000–2015. Addictive Behaviors. 2019 Feb 1;89:51–6.
9. Wulfert E, Harris K, Broussard J. The role of cross-cue reactivity in coexisting smoking and gambling habits. J Gambl Issues. 2016;32:28–43.

For peer review only - http://bmjopen.bmj.com/site/about/guidelines.xhtml

10. McGrath DS, Barrett SP. The comorbidity of tobacco smoking and gambling: A review of the literature. Drug Alcohol Rev [Internet]. 2009 Nov 1 [cited 2023 Jan 10];28(6):676–81. Available from: https://onlinelibrary.wiley.com/doi/full/10.1111/j.1465-3362.2009.00097.x
11. Tackett JL, Krieger H, Neighbors C, Rinker D, Rodriguez L, Edward G. Comorbidity of Alcohol and Gambling Problems in Emerging Adults: A Bifactor Model Conceptualization. J Gambl Stud [Internet]. 2017 Mar 1 [cited 2023 Jan 10];33(1):131–47. Available from: https://link.springer.com/article/10.1007/s10899-016-9618-6
12. Griffiths M, Wardle H, Orford J, Sproston K, Erens B. Gambling, alcohol, consumption, cigarette smoking and health: Findings from the 2007 British Gambling Prevalence Survey. http://dx.doi.org/103109/16066350902928569 [Internet]. 2010 [cited 2023 Jan 6];18(2):208–23. Available from: https://www.tandfonline.com/doi/abs/10.3109/16066350902928569
13. Hahmann T, Hamilton-Wright S, Ziegler C, Matheson FI. Problem gambling within the context of poverty: a scoping review. Int Gambl Stud. 2021;21(2):183–219.
14. Bloomfield K. Understanding the alcohol-harm paradox: what next? Lancet Public Health [Internet]. 2020 Jun 1 [cited 2023 Jun 29];5(6):e300–1. Available from: http://www.thelancet.com/article/S2468266720301195/fulltext
15. Hiscock R, Bauld L, Amos A, Fidler JA, Munafò M. Socioeconomic status and smoking: a review. Ann N Y Acad Sci [Internet]. 2012 Feb [cited 2018 Jun 16];1248(1):107–23. Available from: http://doi.wiley.com/10.1111/j.1749-6632.2011.06202.x
16. Bank of England. How has the increase in the cost of living affected UK households and what does that mean for the economy? [Internet]. Bank Overground. 2022 [cited 2023 Feb 2]. Available from: https://www.bankofengland.co.uk/bank-overground/2022/how-has-the-increase-in-the-cost-of-living-affected-uk-households
17. Kock L, Shahab L, Moore G, Beard E, Bauld L, Reid G, et al. Protocol for expansion of an existing national monthly survey of smoking behaviour and alcohol use in England to Scotland and Wales: The Smoking and Alcohol Toolkit Study. Wellcome Open Res [Internet]. 2021 Mar 29 [cited 2021 Jun 27];6:67. Available from: https://doi.org/10.12688/wellcomeopenres.16700.1
18. Fidler JA, Shahab L, West O, Jarvis MJ, McEwen A, Stapleton JA, et al. "The smoking toolkit study": a national study of smoking and smoking cessation in England. BMC Public Health [Internet]. 2011;11:479. Available from: http://dx.doi.org/10.1186/1471-2458-11-479
19. Ferris JA, Wynne HJ. The Canadian problem gambling index: User manual. Canadian Centre on Substance Abuse; 2001.
20. Holtgraves T. Evaluating the problem gambling severity index. J Gambl Stud [Internet]. 2009 Mar 14 [cited 2023 Jan 12];25(1):105–20. Available from: https://link.springer.com/article/10.1007/s10899-008-9107-7
21. Currie SR, Casey DM, Hodgins DC. Improving the psychometric properties of the Problem Gambling Severity Index. Canadian Consortium for Gambling Research Ottawa; 2010.
22. Babor TF, Higgins-Biddle JC, Saunders JB, Monteiro MG. The alcohol use disorders identification test. World Health Organization Geneva; 2001.
23. NRS. National Readership Survey - Social Grade [Internet]. 2017 [cited 2017 Nov 23]. Available from: http://www.nrs.co.uk/nrs-print/lifestyle-and-classification-data/social-grade/
24. Wickham H, Averick M, Bryan J, Chang W, McGowan LD, François R, et al. Welcome to the Tidyverse. J Open Source Softw. 2019;4(43):1686.
25. Lumley T. Analysis of complex survey samples. J Stat Softw. 2004;9:1–19.

For peer review only - http://bmjopen.bmj.com/site/about/guidelines.xhtml

26. Roberts A, Sharman S, Coid J, Murphy R, Bowden-Jones H, Cowlishaw S, et al. Gambling and negative life events in a nationally representative sample of UK men. Addictive Behaviors. 2017 Dec 1;75:95–102.
27. Huggett SB, Winiger EA, Corley RP, Hewitt JK, Stallings MC. Alcohol use, psychiatric disorders and gambling behaviors: A multi-sample study testing causal relationships via the co-twin control design. Addictive Behaviors. 2019 Jun 1;93:173–9.
28. Thorne HB, Rockloff MJ, Ferguson SA, Vincent GE, Browne M. Gambling Problems Are Associated with Alcohol Misuse and Insomnia: Results from a Representative National Telephone Survey. International Journal of Environmental Research and Public Health 2021, Vol 18, Page 6683 [Internet]. 2021 Jun 22 [cited 2023 Jun 8];18(13):6683. Available from: https://www.mdpi.com/1660-4601/18/13/6683/htm
29. Yi S, Kanetkar V. Coping with Guilt and Shame After Gambling Loss. J Gambl Stud [Internet]. 2011 Sep 21 [cited 2023 Jun 9];27(3):371–87. Available from: https://link.springer.com/article/10.1007/s10899-010-9216-y
30. Zack M, Stewart SH, Klein RM, Loba P, Fragopoulos F. Contingent gambling-drinking patterns and problem drinking severity moderate implicit gambling-alcohol associations in problem gamblers. J Gambl Stud [Internet]. 2005 Sep [cited 2023 Jun 9];21(3):325–54. Available from: https://link.springer.com/article/10.1007/s10899-005-3102-z
31. Horn TL, Whelan JP, Weil GT. Does acute alcohol consumption increase risk-taking while gambling? A systematic review and meta-analysis. Addiction. 2022;117(11):2780–90.
32. McGrath DS, Barrett SP. The comorbidity of tobacco smoking and gambling: a review of the literature. Drug Alcohol Rev. 2009;28(6):676–81.
33. Sharman S. The ability of the UK population surveys to capture the true nature of the extent of gambling related harm. Addiction. 2022;
34. Reuter P, Caulkins JP, Midgette G. Heroin use cannot be measured adequately with a general population survey. Addiction. 2021 Oct 1;116(10):2600–9.
35. Sharman S, Dreyer J, Aitken M, Clark L, Bowden-Jones H. Rates of problematic gambling in a British homeless sample: A preliminary study. J Gambl Stud [Internet]. 2015 Jun 1 [cited 2023 Jun 9];31(2):525–32. Available from: https://link.springer.com/article/10.1007/s10899-014-9444-7
36. Abbott MW. The changing epidemiology of gambling disorder and gambling-related harm: public health implications. Public Health. 2020 Jul 1;184:41–5.
37. UK Government. High stakes: gambling reform for the digital age [Internet]. 2023 [cited 2023 Jul 20]. Available from: https://www.gov.uk/government/publications/high-stakes-gambling-reform-for-the-digital-age/high-stakes-gambling-reform-for-the-digital-age

For peer review only

For peer review only - http://bmjopen.bmj.com/site/about/guidelines.xhtml

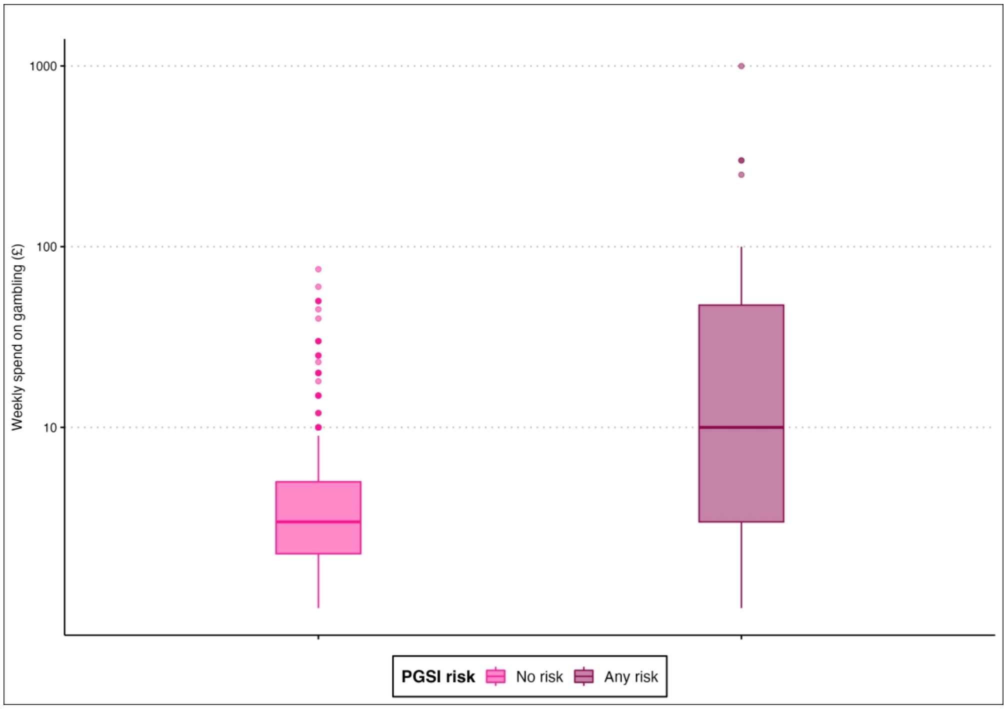

Figure 1: The distribution of mean weekly expenditure (log transformed scale) on gambling according to no risk, or any risk of harm from gambling (scores >0 on the PGSI)

645x452mm (118 x 118 DPI)

Supplementary appendix

Table S1: Types of gambling

Type of gambling	Overall (n=2398)	Smoking (n=345)	Audit-c >4 (n=775)
National lottery, other lotteries of scratch cards	38.4 % (850)	43.8% (138)	45.8% (330)
Football pools	2.1 % (45)	2.3% (8)	4.0% (29)
Online gambling in slots, casino or bingo	4.1% (92)	7.6% (24)	7.9% (57)
Bingo (not online)	3.4% (75)	5.7% (18)	4.8% (35)
Casino table games (not online)	1.7% (39)	2.6% (8)	3.5% (25)
Slot machines	2.8% (62)	6.3% (20)	5.9% (43)
Online betting with bookmaker	5.5% (122)	6.0% (19)	8.0% (58)
Machines in a bookmaker	0.4% (10)	0.0% (0)	0.9% (7)
Horse races (not online)	4.8% (106)	4.9% (16)	7.6% (55)
Dog races (not online)	0.5% (12)	0.3% (1)	0.4% (3)
Sports evens (not online)	1.9% (42)	2.2% (7)	2.8% (20)
Betting exchange	0.4% (9)	0.0% (0)	0.4% (3)
Private betting	0.5% (10)	0.0% (0)	0.6% (4)
Loot boxes or skins gambling within online video games	1.5% (33)	1.9% (6)	1.7% (13)
Crypto casinos	0.0%	0.0% (0)	0.0% (0)
Any other gambling activity	1.6% (36)	1.4% (4)	2.1% (15)

Percentages are weighted

Table S2: Full model output for association between current cigarette smoking, or drinking at increasing and higher risk levels, and any risk gambling according to the PGSI*

Variable	Event Rate	OR	95% CI	P
**Current cigarette smoking**				
**PGSI category**				
No risk	125 / 897 (14%)	—	—	
Any risk	10 / 57 (18%)	0.80	0.35, 1.66	0.57
**Age**				
18-24	22 / 82 (27%)	—	—	
25-34	36 / 142 (25%)	0.94	0.50, 1.82	0.86
35-44	16 / 146 (11%)	0.36	0.17, 0.76	0.007
45-54	23 / 191 (12%)	0.37	0.18, 0.73	0.004
55-64	24 / 204 (12%)	0.33	0.16, 0.64	0.001
65+	14 / 189 (7%)	0.18	0.08, 0.38	<0.001
Sex				
Men	70 / 499 (14%)	—	—	
Women	65 / 455 (14%)	1.01	0.68, 1.49	0.98
**Region**				
South	44 / 310 (14%)	—	—	

For peer review only

Midlands	24 / 217 (11%)	0.75	0.42, 1.29	0.30
North	36 / 229 (16%)	1.05	0.63, 1.73	0.86
Wales	9 / 69 (13%)	1.09	0.46, 2.36	0.83
Scotland	22 / 129 (17%)	1.20	0.66, 2.16	0.54
**Social grade**				
AB	15 / 241 (6%)	—	—	
C1	54 / 427 (13%)	2.06	1.15, 3.89	0.020
C2	30 / 157 (19%)	3.53	1.82, 7.07	<0.001
D	18 / 69 (26%)	5.15	2.37, 11.3	<0.001
E	18 / 60 (30%)	8.14	3.66, 18.4	<0.001
**Drinking at increasing and higher risk levels**				
**PGSI category**				
No risk	363 / 885 (41%)	—	—	
Any risk	26 / 58 (45%)	0.94	0.52, 1.69	0.83
**Age**				
18-24	48 / 80 (60%)	—	—	
25-34	54 / 140 (39%)	0.42	0.23, 0.76	0.004
35-44	72 / 146 (49%)	0.62	0.35, 1.11	0.11
45-54	75 / 190 (39%)	0.44	0.25, 0.76	0.004
55-64	90 / 202 (45%)	0.54	0.31, 0.94	0.029
65+	50 / 185 (27%)	0.26	0.14, 0.46	<0.001
**Sex**				
Men	247 / 491 (50%)	—	—	
Women	142 / 452 (31%)	0.45	0.34, 0.59	<0.001
**Region**				
South	115 / 304 (38%)	—	—	
Midlands	85 / 213 (40%)	1.14	0.78, 1.67	0.49
North	110 / 235 (47%)	1.63	1.13, 2.35	0.010
Wales	22 / 66 (33%)	0.89	0.49, 1.59	0.70
Scotland	57 / 125 (46%)	1.40	0.89, 2.19	0.14
**Social grade**				
AB	102 / 241 (42%)	—	—	
C1	182 / 421 (43%)	1.04	0.75, 1.46	0.81
C2	73 / 153 (48%)	1.14	0.74, 1.75	0.55
D	27 / 69 (39%)	0.83	0.46, 1.46	0.51
E	5 / 59 (9%)	0.13	0.04, 0.32	<0.001

For peer review only

For peer review only

STROBE 2007 (v4) Statement—Checklist of items that should be included in reports of *cross-sectional studies*

Section/Topic	Item #	Recommendation	Reported on page #
**Title and abstract**	1	(*a*) Indicate the study's design with a commonly used term in the title or the abstract	1
		(*b*) Provide in the abstract an informative and balanced summary of what was done and what was found	1
**Introduction**			
Background/rationale	2	Explain the scientific background and rationale for the investigation being reported	3
Objectives	3	State specific objectives, including any prespecified hypotheses	3
**Methods**			
Study design	4	Present key elements of study design early in the paper	4-6
Setting	5	Describe the setting, locations, and relevant dates, including periods of recruitment, exposure, follow-up, and data collection	4-6
Participants	6	(*a*) Give the eligibility criteria, and the sources and methods of selection of participants	4
Variables	7	Clearly define all outcomes, exposures, predictors, potential confounders, and effect modifiers. Give diagnostic criteria, if applicable	4-6
Data sources/ measurement	8*	For each variable of interest, give sources of data and details of methods of assessment (measurement). Describe comparability of assessment methods if there is more than one group	4-6
Bias	9	Describe any efforts to address potential sources of bias	*4*
Study size	10	Explain how the study size was arrived at	4
Quantitative variables	11	Explain how quantitative variables were handled in the analyses. If applicable, describe which groupings were chosen and why	4-6
Statistical methods	12	(*a*) Describe all statistical methods, including those used to control for confounding	6
		(*b*) Describe any methods used to examine subgroups and interactions	NA
		(*c*) Explain how missing data were addressed	6
		(*d*) If applicable, describe analytical methods taking account of sampling strategy	6
		(*e*) Describe any sensitivity analyses	7
**Results**			

For peer review only

Participants	13*	(a) Report numbers of individuals at each stage of study—eg numbers potentially eligible, examined for eligibility, confirmed eligible, included in the study, completing follow-up, and analysed	7-8
		(b) Give reasons for non-participation at each stage	NA
		(c) Consider use of a flow diagram	NA
Descriptive data	14*	(a) Give characteristics of study participants (eg demographic, clinical, social) and information on exposures and potential confounders	7-8
		(b) Indicate number of participants with missing data for each variable of interest	7-8
Outcome data	15*	Report numbers of outcome events or summary measures	7-8
Main results	16	(*a*) Give unadjusted estimates and, if applicable, confounder-adjusted estimates and their precision (eg, 95% confidence interval). Make clear which confounders were adjusted for and why they were included	7-9
		(*b*) Report category boundaries when continuous variables were categorized	7-10
		(*c*) If relevant, consider translating estimates of relative risk into absolute risk for a meaningful time period	NA
Other analyses	17	Report other analyses done—eg analyses of subgroups and interactions, and sensitivity analyses	7-10
**Discussion**			
Key results	18	Summarise key results with reference to study objectives	10-11
Limitations	19	Discuss limitations of the study, taking into account sources of potential bias or imprecision. Discuss both direction and magnitude of any potential bias	10-11
Interpretation	20	Give a cautious overall interpretation of results considering objectives, limitations, multiplicity of analyses, results from similar studies, and other relevant evidence	10-11
Generalisability	21	Discuss the generalisability (external validity) of the study results	10-11
**Other information**			
Funding	22	Give the source of funding and the role of the funders for the present study and, if applicable, for the original study on which the present article is based	11

*Give information separately for cases and controls in case-control studies and, if applicable, for exposed and unexposed groups in cohort and cross-sectional studies.

Note: An Explanation and Elaboration article discusses each checklist item and gives methodological background and published examples of transparent reporting. The STROBE checklist is best used in conjunction with this article (freely available on the Web sites of PLoS Medicine at http://www.plosmedicine.org/, Annals of Internal Medicine at http://www.annals.org/, and Epidemiology at http://www.epidem.com/). Information on the STROBE Initiative is available at www.strobe-statement.org.

For peer review only

BMJ Open

The Intersection of Gambling with Smoking and Alcohol use in Great Britain: A Cross-Sectional Survey in October 2022

Journal:	*BMJ Open*
Manuscript ID	bmjopen-2023-079633.R1
Article Type:	Original research
Date Submitted by the Author:	02-Mar-2024
Complete List of Authors:	Kock, Loren; University College London Research Department of Epidemiology and Public Health, Behavioural Science and Health; Dept. Behavioural Science & Health<br>Cox, Sharon; University College London, Behavioural Science and Health; SPECTRUM Research Consortium,<br>Shahab, Lion; UCL, Dept of Epidemiology and Public Health<br>Roberts, Amanda; University of Lincoln<br>Sharman, Steve; King's College London,<br>Buss, Vera; University College London<br>Brown, Jamie; University College London, Psychology & Language Sciences
**Primary Subject Heading**:	Addiction
Secondary Subject Heading:	Public health, Epidemiology
Keywords:	Behavior, EPIDEMIOLOGY, Substance misuse < PSYCHIATRY

SCHOLARONE™
Manuscripts

I, the Submitting Author has the right to grant and does grant on behalf of all authors of the Work (as defined in the below author licence), an exclusive licence and/or a non-exclusive licence for contributions from authors who are: i) UK Crown employees; ii) where BMJ has agreed a CC-BY licence shall apply, and/or iii) in accordance with the terms applicable for US Federal Government officers or employees acting as part of their official duties; on a worldwide, perpetual, irrevocable, royalty-free basis to BMJ Publishing Group Ltd ("BMJ") its licensees and where the relevant Journal is co-owned by BMJ to the co-owners of the Journal, to publish the Work in this journal and any other BMJ products and to exploit all rights, as set out in our licence.

The Submitting Author accepts and understands that any supply made under these terms is made by BMJ to the Submitting Author unless you are acting as an employee on behalf of your employer or a postgraduate student of an affiliated institution which is paying any applicable article publishing charge ("APC") for Open Access articles. Where the Submitting Author wishes to make the Work available on an Open Access basis (and intends to pay the relevant APC), the terms of reuse of such Open Access shall be governed by a Creative Commons licence – details of these licences and which Creative Commons licence will apply to this Work are set out in our licence referred to above.

Other than as permitted in any relevant BMJ Author's Self Archiving Policies, I confirm this Work has not been accepted for publication elsewhere, is not being considered for publication elsewhere and does not duplicate material already published. I confirm all authors consent to publication of this Work and authorise the granting of this licence.

For peer review only

The Intersection of Gambling with Smoking and Alcohol use in Great Britain: A Cross-Sectional Survey in October 2022

Loren Kock[1,2], Sharon Cox[1,2], Lion Shahab[1,2], Amanda Roberts[3], Steve Sharman[4] Vera Buss[1,2], Jamie Brown[1,2]

[1]Department of Behavioural Science and Health, University College London, UK
[2]SPECTRUM Research Consortium, Edinburgh, UK
[3]School of Psychology, University of Lincoln, UK
[4]National Addiction Centre, King's College London, UK

Correspondence to: Dr Loren Kock (l.kock@ucl.ac.uk)

Word count:

ABSTRACT

Objectives: Gambling is associated with cigarette smoking and alcohol consumption. We explored the intersection of gambling across all risk levels of harm with smoking and alcohol use among adults in Great Britain.

Design: A nationally representative cross-sectional survey in October 2022.

Setting: Great Britain.

Participants: A weighted total of 2,398 adults (18+ years).

Outcome measures: We examined the prevalence of past-year gambling and, among those reporting gambling, assessed the associations between the outcome of any risk of harm from gambling (scoring >0 on the problem gambling severity index) and the binary predictor variables of current cigarette smoking and higher risk alcohol consumption (AUDIT-C ≥4). We also explored data on weekly expenditure on gambling with smoking and alcohol use among those categorised at any-risk of harm from gambling.

Results: Overall, 43.6% (95% CI 41.2-45.9) of adults gambled in the past year. Among these, 7.3% (5.3-9.3) were classified at any-risk of harm from gambling, 16.0% (13.2-18.8) were currently smoking, and 40.8% (37.2-44.4) were drinking at increasing and higher risk levels. There were no associations between any risk of harm from gambling and current smoking (ORadj=0.80, 95% CI 0.35-1.66) or drinking at increasing and higher risk levels (ORadj=0.94, 0.52-1.69), respectively. Analyses using Bayes factors indicated that these data were insensitive to distinguish no effect from a range of associations (OR=0.5-1.9). The mean weekly spend on gambling was £7.69 (95% CI 5.17-10.21) overall, £4.80 (95% CI 4.18-5.43) among those classified as at no risk, and £45.68 (12.07-79.29) among those at any risk of harm from gambling.

Conclusions: Pilot data in a population-level survey on smoking and alcohol use yielded similar estimates to other population-level surveys on gambling participation and at-risk gambling. Further data are needed to elucidate the intersections more reliably between gambling, smoking and alcohol use, and inform population-level approaches to reduce harm.

Keywords: Disordered gambling, smoking, alcohol use

For peer review only - http://bmjopen.bmj.com/site/about/guidelines.xhtml

Strengths and limitations of the study

- The main strength of this study is the inclusion of measures of gambling behavior in a long-running representative survey of the Smoking and Alcohol use in Great Britain.
- Limitations are that the data are cross-sectional and self-reported, and a small number of respondents were classified as at any-risk of harm from gambling.
- There is uncertainty in our estimates for expenditure on gambling among those at any risk of harm from gambling, but the upper limits remain plausible for disordered gambling behavior.

For peer review only

INTRODUCTION

General population surveys estimate that half of adults in Great Britain have gambled in the previous 12 months [1,2]. Although many individuals gamble as a social activity without experiencing harm, some gamble at levels that adversely impact financial stability, personal and family wellbeing and physical and mental health [3,4]. Conservative estimates indicate that approximately 0.3-0.5% of the general UK adult population report severe gambling behaviours that warrant a diagnosis of gambling disorder (hereafter termed "disordered gambling") and 3-4% are "at-risk" (those who experience a low or moderate level of problems leading to some negative consequences, and relative to disordered gamblers drive most of the harm from gambling at the population level) [2,5–7]. Due to a combination of financial and health costs associated with gambling (including homelessness, suicide, depression, alcohol dependence, illicit substance use, unemployment and imprisonment), gambling is also associated with an estimated annual economic cost to the UK government of ~£413 million, and potentially £655-£1,355 million in societal value due to adverse health effects [8].

Gambling is thought to be associated with other addictive behaviours, including cigarette smoking and excess substance use such as alcohol consumption [9–11], and this may relate to common neurobiological, genetic and socio-environmental factors which could act to reinforce each respective behaviour [12–14]. Previous prevalence surveys in Great Britain have illustrated mixed evidence on the relationship between disordered gambling and use of these substances. The 2007 British Gambling survey indicated that smoking was associated with higher rates of past-year gambling (79% vs 64% in those who did not smoke) and disordered gambling (1.4% vs [15]0.4%), while the prevalence of disordered gambling was 3.4% in those who consumed >20 units of alcohol on their heaviest drinking day and 0.1% who consumed 1-4 units. [16]. In contrast, a 2021 evidence review on gambling related harms conducted by Public Health England concluded that although increased alcohol consumption was associated with gambling at all levels of harm, there was no apparent association with smoking [2].

The co-occurrence of gambling with smoking and increasing and higher risk alcohol consumption is important to study at the population level in the context of public health and health inequalities. It is likely that co-occurrence of these behaviours compound the physical, social, financial and psychological harms that each of them cause. These harms may be disproportionately greater for certain sub-groups, namely those experiencing poverty [17] who are also more likely to smoke and experience greater harms from alcohol consumption compared with more advantaged groups [18,19]. Due to their high relative costs, expenditure on smoking and alcohol can exacerbate and push low-income households into poverty [15]. Likewise, money spent on gambling as a proportion of total expenditure may be higher in less advantaged households [20]. Expenditure on all three products is of concern particularly in the UK and elsewhere where rising prices for everyday items and services have resulted in less 'disposable' income, particularly among lower income households [21]. Since these individuals are less able to absorb the added burden of this 'cost of living' crisis, it follows that less advantaged groups suffer greater psychosocial and material harm than more advantaged households even in the absence of the harms caused by gambling and substance use behaviours [22].

For peer review only - http://bmjopen.bmj.com/site/about/guidelines.xhtml

Smoking, alcohol and gambling among adults aged 18+ is currently legal in the UK, with the highly profitable underlying industries regulated to different degrees by the UK government but with similar motives to disrupt policies seeking to reduce the harm from use of their products [23]. Data from a representative sample of adults can provide insight into the dynamics of these behaviours – for instance the potential to substitute or complement one with another [24,25] – in an evolving sociocultural and regulatory context. To build on the existing research reporting on these three behaviours, we piloted the addition of several standard gambling measures to an ongoing representative monthly survey of smoking and alcohol use in Great Britain. The objective of this study is to explore the intersection of gambling across all risk levels (henceforth termed "any-risk" gambling) with smoking and alcohol use among adults in Great Britain. Specifically, we aimed to i) estimate the prevalence of past-year gambling according to smoking and increasing and higher risk alcohol use, ii) assess the associations between any-risk gambling (defined by scores of >0 on the problem gambling severity index) and smoking and increasing and higher risk alcohol use, respectively and iii) explore the average weekly expenditure on gambling, smoking and alcohol use among those reporting any-risk gambling.

METHODS

Sample and recruitment

The study population consisted of adults aged ≥18 and over living in households in Great Britain, surveyed in October 2022 in the Smoking and Alcohol Toolkit Study (STS/ATS). Ethical approval for the STS/ATS was granted by the UCL Ethics Committee (ID 0498/001). The data were not collected by UCL and were anonymised when received by the research team. In accordance with ethical approval, all respondents provided informed verbal consent.

The STS/ATS uses a hybrid of random location and quota sampling to select a new sample of approximately 2,400 adults (aged ≥16 years) each month in Great Britain. Telephone interviews are carried out with one household member until quotas based on factors influencing the probability of being at home (e.g., gender, age, working status) are fulfilled. We used survey weighting to match descriptive data to sociodemographic profile in Great Britain (based on age, social grade, region, tenure, ethnicity and working status within sex). Detailed survey methodology is reported elsewhere [26,27]. Comparisons with sales data and other national surveys show that the STS recruits a representative sample of the population in Great Britain with regard to key demographic variables and smoking indicators.

For the current study, all adults were asked a question pertaining to past year gambling participation (derived from indications of type of gambling). Due to funding constraints, questions used to derive the Problem Gambling Severity Index and weekly expenditure on gambling were asked to a partial sample consisting of ~88% of the total eligible sample of people who indicated that they gambled in the past year.

Measures

The measures included in the current study are summarized in Table 1 below. Full details on items used to code these variables are provided in the supplementary appendix.

Table 1: Summary of measures

For peer review only - http://bmjopen.bmj.com/site/about/guidelines.xhtml

Measure	***Description***	***Variable type***
**Past-year gambling** (Categorical)<br><br>Levels:<br>Yes; No)	Affirmative responses to any of the below gambling types in the past year:<br><br>• National lottery, other lotteries, or scratch cards<br>• Football pools<br>• Bingo (not online)<br>• Slot machines<br>• Machines in a bookmakers<br>• Casino table games (not online)<br>• Online gambling in slots, casino, or bingo<br>• Online betting with a bookmaker<br>• Betting exchange<br>• Horse races (not online)<br>• Dog races (not online)<br>• Sports events (not online)<br>• Private betting<br>• Loot boxes or skins gambling within online/video games<br>• Crypto casinos<br>• Any other gambling event or activity<br>• Have not done any of these things	Binary stratification variable
**Problem gambling severity index** (categorical)<br><br>Levels:<br>No risk; Low risk;<br>Moderate risk;<br>Disordered gambling	The PGSI is a nine item questionnaire on gambling severity and was derived from the Canadian Problem Gambling Index [28,29], and asked to those categorized as having gambled in the past year (see Appendix for full item list).<br><br>Scores between 1 and 7 represent "at risk" gambling (one to four "low risk" and five to seven "moderate risk"). An individual scoring eight or higher is classified as a "disordered gambler" [30].	Outcome
Any risk of harm from gambling (categorical)<br><br>Levels:<br>No risk; Any risk	A category of "any-risk" refers to those scoring 1 or greater on the PGSI.	Outcome (recode)

For peer review only - http://bmjopen.bmj.com/site/about/guidelines.xhtml

Weekly expenditure on gambling, smoking and/or alcohol (continuous: in GBP (£))	Weekly expenditure on gambling, smoking and alcohol was derived from responses to three questions regarding expenditure on each.	Outcome
Smoking status (categorical)<br><br>Levels:<br>Currently smoking; Not smoking	Respondents were classified according to whether they were currently smoking cigarettes (Smoke every day; Smoke but not every day) or not (Do not smoke cigarettes but smoke tobacco of some kind; Stopped smoking in the last year; Stopped smoking more than 1 year ago; never smoked).	Predictor
Level of alcohol consumption (categorical)<br><br>Levels:<br><4 on AUDIT-C;<br>≥4 on AUDIT-C (drinking at increasing and higher risk levels)	Level of alcohol consumption in the last six months was assessed using the consumption items from the Alcohol Use Disorders Identification Test (AUDIT-C) [25], a three-item screening tool developed by the World Health Organisation, with a score ranging from 0 to 12.<br><br>Respondents scoring 4 or higher on the AUDIT-C were classified as drinking alcohol at increasing and high-risk levels.	Predictor
Social grade (categorical)<br><br>Levels:<br>ABC1; C2DE	Social grade based on occupation (ABC1: higher and intermediate managerial, administrative, and professional, supervisory, clerical and junior managerial, administrative and professional; C2DE: skilled manual workers, semi-skilled and unskilled manual workers and state pensioners, casual and lowest-grade workers, unemployed with state benefits)[31].	Covariate
Age (categorical)<br><br>Levels:<br>18-24; 25-34; 35-44; 45-54; 65+	Age in years at the time of the survey	Covariate
Sex (categorical)<br><br>Levels:<br>Women; Men; In another way/refused	Identified sex at the time of the survey<br><br>All response options were reported in sample characteristics, but due to small case numbers of “in another way/refused” this category was excluded from regression analyses), and	Covariate

For peer review only

For peer review only - http://bmjopen.bmj.com/site/about/guidelines.xhtml

Region in GB (categorical)<br><br>Levels:<br>North, Midlands, South Scotland; Wales	Region in England at time of the survey.	Covariate

Patient and public involvement
None

Analyses

The analyses were pre-registered on the open science framework, https://osf.io/nc6jm and conducted in R version 4.2.2 (packages *tidyverse* and *survey* [32,33]) with all statistical code made open-access at https://osf.io/aj7c9/. The study followed the Strengthening the Reporting of Observational Studies in Epidemiology (STROBE) guidelines for cross-sectional studies.

Characteristics of the sample and descriptive statistics are presented using weighted descriptive statistics. Under the first study aim, the prevalence of past-year gambling and any-risk gambling (according to the PGSI) are presented weighted with 95% confidence intervals. Estimates are reported both as a percentage of the overall population, and of those who gambled in the past-year (in the case of any-risk gambling).

Under the second study aim, we constructed logistic regression models to assess the associations between any-risk gambling (reference group: no risk) with current smoking (reference group: not smoking), increasing and higher risk alcohol consumption (reference group: <4 on AUDIT-C), respectively among people who gambled in the past year. All models are adjusted for key sociodemographic characteristics (age, sex, social grade, and region). Respondents with missing data on any of the covariates of interest (3.4% of total sample) were excluded from the analyses.

Under the third study aim, we estimated the average weekly expenditure on i) gambling and ii) gambling, smoking and alcohol use, among those classified as "any-risk" compared with those who gamble without risk. These data are presented descriptively as mean expenditure with measures of spread (standard error, 95% CI and range).

Unregistered changes to analysis plan

Observed non-significant associations between any risk of gambling harm and current smoking, or drinking at increasing and higher risk levels could have indicated evidence for no association, or that the data were insensitive to detect an effect. To explore this, post-hoc Bayes factors are calculated for a range of hypothetical effect sizes including the potential for lower odds (OR=0.5 or 0.9) or higher odds (OR=1.1, 1.5, or 1.9) of any risk of gambling harm according to cigarette smoking and AUDIT-C scores of four or more, respectively. Bayes factors were computed using an online calculator (www.bayesfactor.info).

RESULTS

For peer review only - http://bmjopen.bmj.com/site/about/guidelines.xhtml

A weighted total of 2,398 adults aged 18 and older (mean [SE] age = 47.7 [0.46]) surveyed in October 2022 were included in the analytic sample (Table 2). In the overall sample, 43.6% (95% CI 41.2-45.9) of adults participated in a gambling activity in the past year, and 3.2% (2.3-4.1) were classified as having any-risk of harm from gambling (i.e., scoring >0 on the PGSI), 14.5% (12.8-16.3) were currently smoking, and 33.4% (31.2-35.7) were drinking at increasing and higher risk levels. Among those who reported any gambling activity in the past year (n=1,045), 7.3% (5.3-9.3) were classified as being at any-risk of harm from gambling with 0.3% (0.0-0.66) classified as disordered gambling, 16.0% (13.2-18.8) were currently smoking, and 40.8% (37.2-44.4) were drinking at increasing and higher risk levels (Table 2). Aside from gambling on the national lottery, other lotteries or scratch cards (38.4%), the three most common gambling activities overall were online betting with bookmaker (5.5%), horse races (not online) (4.8%) and online gambling in slots, casino or bingo (4.1%) (Table S1).

In the models adjusting for age, sex, social grade and region, there were no apparent associations between any risk of harm from gambling and current cigarette smoking (ORadj=0.80, 95% CI 0.35-1.66) or drinking at increasing and higher risk levels (ORadj=0.94, 95% CI 0.52-1.69), respectively (Table 3 and Table S2). Analyses using Bayes factors indicated that the data were insensitive to detect an effect in either direction, and therefore these results are inconclusive (Table S3).

In the sample of adults who gambled in the past year, the mean weekly spend on gambling was £4.80 (95% CI 4.18-5.43) among those classified as at no risk, and £45.68 (12.07-79.29) among those classified as at any risk of harm from gambling according to the PGSI (Figure 1 and Table S4). Caution should be taken in the interpretation of expenditure in the any risk category due to a relatively small number of cases (n=67) compared with the no risk category (n=878). The distribution of mean weekly spend on gambling is shown in Figure S1 and highlights how the mean is influenced by a small number of higher values in the any risk category (one respondent reported a weekly mean spend on gambling of £998.00). The equivalent expenditure in a sample excluding those who only gambled on lottery/scratch cards was £6.42 (4.99-7.87) in those at no risk and £56.48 (14.79-98.17) in those at any risk of harm from gambling. The mean weekly spend on gambling was £8.09 (3.52-12.65) in people currently smoking (vs. £7.61 in those not smoking) and £10.74 (4.86-16.66) among people drinking at increasing and higher risk levels (vs. £5.26 in people with AUDIT-C scores of< 4), respectively (Figure 1 and Table S4). Overall, among those who smoked or were drinking at increasing and higher risk levels, spend on gambling and smoking was £42.73 (33.88-51.59.), gambling and alcohol was £36.48 (26.83-46.13), and on all three behaviours was £69.37 (48.78-89.96) (Table S4).

Table 2: Characteristics of sample

	**Overall**	**Did not gamble**	**Gambled in past year**
Characteristic	**n = 2,398**	**n = 1,353**	**n = 1,045**
**Age**			
18-24	13.7%, (328)	16.2%, (219)	10.5%, (110)
25-34	16.7%, (400)	16.3%, (221)	17.2%, (180)
35-44	15.4%, (370)	15.0%, (203)	16.0%, (168)
45-54	16.5%, (395)	14.8%, (201)	18.6%, (194)
55-64	15.0%, (359)	12.1%, (164)	18.6%, (194)
65+	22.7%, (544)	25.5%, (345)	19.1%, (199)
Missing	2	2	0

For peer review only - http://bmjopen.bmj.com/site/about/guidelines.xhtml

**Sex**			
Men	48.7%, (1,157)	46.5%, (622)	51.5%, (535)
Women	50.8%, (1,209)	53.1%, (710)	47.9%, (499)
In another way	0.5%, (12)	0.4%, (6)	0.6%, (6)
Missing	20	15	5
**Social grade**			
AB	26.1%, (626)	26.4%, (357)	25.8%, (269)
C1	29.9%, (716)	29.5%, (399)	30.3%, (316)
C2	20.3%, (486)	19.9%, (270)	20.7%, (216)
D	14.5%, (348)	14.2%, (193)	14.8%, (156)
E	9.3%, (222)	9.9%, (134)	8.4%, (88)
**Region**			
South	36.6%, (878)	36.1%, (489.2)	37.2%, (388.3)
Midlands	25.9%, (622)	25.5%, (345.7)	26.5%, (276.5)
North	23.8%, (571)	20.6%, (279.3)	27.9%, (291.6)
Wales	4.9%, (118)	6.2%, (84.0)	3.3%, (34.1)
Scotland	8.7%, (209)	11.5%, (155.2)	5.2%, (54.1)
**PGSI category**			
Did not gamble	56.4%, (1,353)	100.0%, (1,353)	-
No risk	40.4%, (969)	-	92.7%, (968.5)
Low risk	2.6%, (62)	-	5.9%, (62)
Moderate risk	0.5%, (11)	-	1.0%, (11)
Disordered gambling	0.1%, (3)	-	0.3%, (3)
**Smoked cigarettes**	14.5%, (345)	13.4%, (181)	16.0%, (164)
Missing	26	8	18
**AUDIT-C 4 or higher**			
4 or higher	33.4%, (775)	27.7%, (359)	40.8%, (415)
Missing	82	55	27

Table 3: Association between current cigarette smoking, or drinking at increasing and higher risk levels, and any risk gambling according to the PGSI*

Variable	Event Rate	OR	95% CI	P
**Current cigarette smoking**				
**PGSI category**				
No risk	125 / 897 (14%)	—	—	
Any risk	10 / 57 (18%)	0.80	0.35, 1.66	0.57
**Drinking at increasing and higher risk levels**				
**PGSI category**				
No risk	363 / 885 (41%)	—	—	

For peer review only

For peer review only - http://bmjopen.bmj.com/site/about/guidelines.xhtml

Any risk	26 / 58 (45%)	0.94	0.52, 1.69	0.83

Model adjusted for age, sex, social grade and region.

Figure 1: Mean weekly expenditure on gambling according to PGSI category (A), smoking status (B), and AUDIT-C score (C), and mean weekly expenditure on gambling, smoking, and alcohol (D), gambling and smoking (E) and gambling and alcohol (F) according to PGSI category.

Error bars represent 95% CIs for estimates of mean weekly expenditure

DISCUSSION

In a nationally representative survey of smoking and alcohol use in Great Britain, our pilot gambling questions collected during one month of data collection returned similar estimates for overall gambling participation, and for at-risk and disordered gambling as other national population surveys [1,8]. Approximately half of adults reported some gambling activity in the past year, and descriptively the prevalence of smoking (16%) and increasing and higher risk drinking (41%) was higher in those who gambled compared with those who did not (13% and 28%, respectively).

One in 14 people who gambled were classified as being at any risk of harm from gambling, but our data were likely insensitive to detect associations between any-risk of harm from gambling, smoking and higher risk drinking, if true associations ranging from OR=0.5 to 1.9 existed. Although gambling at any level of harm is consistently associated with alcohol consumption [2,34], the aetiology of this relationship is likely multidimensional. Observed associations in the wider literature may reflect common underlying genetic, social and environmental determinants [35,36], but also involve bi-directional causality whereby frequency of gambling is higher under the influence of alcohol [35]. Alcohol may be used as an avoidant coping mechanism following losses [37] and as a cued response following wins [38]. The mechanism through which alcohol consumption might lead to disordered gambling requires more research. For example, a recent review examining the salient hypothesis that acute alcohol consumption leads to harm from gambling by encouraging greater risk taking concluded that there was no reliable effect [39].

In 2009 a review into the comorbidity of smoking and gambling concluded that comorbidity was highly prevalent [40]. However, an evidence review published in 2023 conducted by Public Health England concluded that cigarette smoking was not associated with gambling among adults [2]. While this may not hold true in certain priority subgroups, further data examining this issue in the STS/ATS could provide important information at the population-level.

Limitations of this study include the data being cross-sectional and self-reported, and the uncertainty in our estimate for prevalance of any-risk of harm from gambling due to the relatively small number of respondents classified into this group. The paucity of data in our survey on

For peer review only

individuals experiencing disordered gambling reflects the relatively small proportion of the population falling into this category, but also that population surveys cannot comprehensively capture relatively rare behaviours - like disordered gambling [41], or injecting drug use [42] – which are more common in subgroups of the population who fall outside of traditional sampling frames [43]. Nonetheless, because they are more numerous, the majority of harms from gambling at the population-level is driven by those classified as low, and moderate risk of harm [5]. Understanding the relationships between gambling behavior and other licit and commercially influenced addictive behaviors like smoking and alcohol use, and gambling at all levels of risk remains an important endeavour. While pilot data were collected in one survey month, extending data collection in a longer monthly time-series would allow these intersections to be interrogated with greater accuracy and reliability.

Finally, while there was wide uncertainty in our estimates for expenditure on gambling among those at any risk of harm from gambling, the outliers driving this uncertainty remain plausible given the extreme spending that can occur in those experiencing disordered gambling [5]. Indeed, due to the rise in online gambling in recent years, in their recent white paper the UK government has proposed introducing financial risk-checks for moderate to high spending [44]. While our estimates should be interpreted with caution, there was a signal for higher expenditure on gambling among those catagorised as drinking at an increasing and higher risk level. If true, this pattern of spending would conform to studies highlighting a positive relationship between increasing alcohol consumption and gambling spend [9].

In conclusion, the collection of pilot data on gambling in a population-level survey on smoking and alcohol use yielded estimates of gambling participation and at-risk and disordered gambling that are similar to other population-level surveys. Further data collection would help elucidate the intersections more reliably between gambling, smoking and alcohol use, and inform population-level approaches to reduce the harms to public health conferred by these behaviors.

Funding

Funding for STS/ATS data collection is supported by Cancer Research UK (PRCRPG-Nov21\100002), and the UK Prevention Research Partnership (MR/S037519/1).

Competing interest statement

JB reports receiving grants from Cancer Research UK during the conduct of the study and receiving unrestricted research funding from pharmaceutical companies who manufacture smoking cessation medications to study smoking cessation outside the submitted work. LS reports receiving honoraria for talks, receiving an unrestricted research grant and travel expenses to attend meetings and workshops by pharmaceutical companies that make smoking cessation products (Pfizer and Johnson & Johnson), and acting as a paid reviewer for grant-awarding bodies and as a paid consultant for health care companies. SC has provided expert consultancy to providers of UK life insurance and the pharmaceutical industry on matters relating to smoking cessation aids. SS has received funding from the Society for the Study of Addiction (SSA), and the NIHR. He is currently employed at the National Addictions Centre, part of the NIHR Biomedical Research Centre and declares no conflicts. AR has received funding from Santander, Public Health for Lincoln, The Royal Society, The Maurice and Jacqueline Bennett Charitable Trust, East Midlands RDS and internal University of Lincoln awards and declares no conflicts of interest. LK, VB and EP have no conflicts of interests to declare.

For peer review only - http://bmjopen.bmj.com/site/about/guidelines.xhtml

Contributors: LK had full access to all of the data in the study and takes responsibility for the integrity of the data and the accuracy of the data analysis. LK was in charge of acquisition, analysis and interpretation of data, drafting of the manuscript, statistical analysis. All authors (LK, JB, SC, LS, VB, SS and AR) were involved in the concept and design of the study. LK led the revision of the manuscript, and all authors were involved in the revision of the manuscript for important intellectual content. LK and JB obtained funding, and JB was LK's supervisor.

Data sharing statement

The analyses were pre-registered on the open science framework, https://osf.io/nc6jm and conducted in R version 4.2.2 (packages *tidyverse* and *survey* [32,33]) with all statistical code made open-access at https://osf.io/aj7c9/. Data are available from authors upon request.

Ethics approval

Ethical approval for the STS/ATS was granted by the UCL Ethics Committee (ID 0498/001). The data were not collected by UCL and were anonymised when received by the research team. In accordance with ethical approval, all respondents provided informed verbal consent.

REFERENCES

1. Conolly A, Davies B, Fuller E, Heinze N, Wardle H. Gambling behaviour in Great Britain in 2016 Evidence from England, Scotland and Wales [Internet]. 2018 [cited 2023 Jan 2]. Available from: https://assets.ctfassets.net/j16ev64qyf6l/60qlzeoSZIJ2QxByMAGJqz/e3af209d552b08c16566a217ed422e68/Gambling-behaviour-in-Great-Britain-2016.pdf
2. Public Health England. Gambling-related harms evidence review: summary [Internet]. 2021 [cited 2023 Jan 3]. Available from: https://www.gov.uk/government/publications/gambling-related-harms-evidence-review/gambling-related-harms-evidence-review-summary
3. Hodgins DC, Stea JN, Grant JE. Gambling disorders. The Lancet. 2011 Nov 26;378(9806):1874–84.
4. Petry NM, Zajac K, Ginley MK. Behavioral Addictions as Mental Disorders: To Be or Not To Be? Annu Rev Clin Psychol [Internet]. 2018 May 5 [cited 2023 Feb 2];14:399. Available from: /pmc/articles/PMC5992581/
5. Abbott MW. The changing epidemiology of gambling disorder and gambling-related harm: public health implications. Public Health. 2020 Jul 1;184:41–5.
6. Gambling Commission. Statistics on participation and problem gambling for the year to March 2023 [Internet]. 2023 [cited 2023 Jun 29]. Available from: https://www.gamblingcommission.gov.uk/statistics-and-research/publication/statistics-on-participation-and-problem-gambling-for-the-year-to-march-2023
7. Browne M, Rockloff MJ. Prevalence of gambling-related harm provides evidence for the prevention paradox. J Behav Addict [Internet]. 2018 Jun 1 [cited 2024 Feb 24];7(2):410–22. Available from: https://akjournals.com/view/journals/2006/7/2/article-p410.xml
8. Office for Health Improvement and Disparities. The economic cost of gambling-related harm in England: evidence update 2023. 2023.
9. Díaz A, Pérez L. Gambling and substance use: A cross-consumption analysis of tobacco smoking, alcohol drinking and gambling. https://doi.org/101080/0889707720211903657

For peer review only - http://bmjopen.bmj.com/site/about/guidelines.xhtml

[Internet]. 2021 [cited 2023 Jan 10];42(4):1016–21. Available from: https://cogentoa.tandfonline.com/doi/abs/10.1080/08897077.2021.1903657
10. Sharman S, Murphy R, Turner JJD, Roberts A. Trends and patterns in UK treatment seeking gamblers: 2000–2015. Addictive Behaviors. 2019 Feb 1;89:51–6.
11. Butler N, Quigg Z, Bates R, Sayle M, Ewart H. Gambling with Your Health: Associations Between Gambling Problem Severity and Health Risk Behaviours, Health and Wellbeing. J Gambl Stud [Internet]. 2020 Jun 1 [cited 2024 Feb 14];36(2):527–38. Available from: https://link.springer.com/article/10.1007/s10899-019-09902-8
12. Wulfert E, Harris K, Broussard J. The role of cross-cue reactivity in coexisting smoking and gambling habits. J Gambl Issues. 2016;32:28–43.
13. McGrath DS, Barrett SP. The comorbidity of tobacco smoking and gambling: A review of the literature. Drug Alcohol Rev [Internet]. 2009 Nov 1 [cited 2023 Jan 10];28(6):676–81. Available from: https://onlinelibrary.wiley.com/doi/full/10.1111/j.1465-3362.2009.00097.x
14. Tackett JL, Krieger H, Neighbors C, Rinker D, Rodriguez L, Edward G. Comorbidity of Alcohol and Gambling Problems in Emerging Adults: A Bifactor Model Conceptualization. J Gambl Stud [Internet]. 2017 Mar 1 [cited 2023 Jan 10];33(1):131–47. Available from: https://link.springer.com/article/10.1007/s10899-016-9618-6
15. Nyakutsikwa B, Britton J, Langley T. The effect of tobacco and alcohol consumption on poverty in the United Kingdom. Addiction [Internet]. 2021 Jan 1 [cited 2024 Feb 14];116(1):150–8. Available from: https://onlinelibrary.wiley.com/doi/full/10.1111/add.15096
16. Griffiths M, Wardle H, Orford J, Sproston K, Erens B. Gambling, alcohol, consumption, cigarette smoking and health: Findings from the 2007 British Gambling Prevalence Survey. http://dx.doi.org/103109/16066350902928569 [Internet]. 2010 [cited 2023 Jan 6];18(2):208–23. Available from: https://www.tandfonline.com/doi/abs/10.3109/16066350902928569
17. Hahmann T, Hamilton-Wright S, Ziegler C, Matheson FI. Problem gambling within the context of poverty: a scoping review. Int Gambl Stud. 2021;21(2):183–219.
18. Bloomfield K. Understanding the alcohol-harm paradox: what next? Lancet Public Health [Internet]. 2020 Jun 1 [cited 2023 Jun 29];5(6):e300–1. Available from: http://www.thelancet.com/article/S2468266720301195/fulltext
19. Hiscock R, Bauld L, Amos A, Fidler JA, Munafò M. Socioeconomic status and smoking: a review. Ann N Y Acad Sci [Internet]. 2012 Feb [cited 2018 Jun 16];1248(1):107–23. Available from: http://doi.wiley.com/10.1111/j.1749-6632.2011.06202.x
20. Hahmann T, Hamilton-Wright S, Ziegler C, Matheson FI. Problem gambling within the context of poverty: a scoping review. Int Gambl Stud [Internet]. 2021 May 4 [cited 2024 Feb 14];21(2):183–219. Available from: https://www.tandfonline.com/doi/abs/10.1080/14459795.2020.1819365
21. Bank of England. How has the increase in the cost of living affected UK households and what does that mean for the economy? [Internet]. Bank Overground. 2022 [cited 2023 Feb 2]. Available from: https://www.bankofengland.co.uk/bank-overground/2022/how-has-the-increase-in-the-cost-of-living-affected-uk-households
22. Broadbent P, Thomson R, Kopasker D, McCartney G, Meier P, Richiardi M, et al. The public health implications of the cost-of-living crisis: outlining mechanisms and modelling consequences. The Lancet Regional Health - Europe [Internet]. 2023 Apr 1 [cited 2024

For peer review only - http://bmjopen.bmj.com/site/about/guidelines.xhtml

Feb 14];27. Available from: http://www.thelancet.com/article/S2666776223000030/fulltext

23. van Schalkwyk MCI, Petticrew M, Cassidy R, Adams P, McKee M, Reynolds J, et al. A public health approach to gambling regulation: countering powerful influences. Lancet Public Health [Internet]. 2021 Aug 1 [cited 2024 Feb 12];6(8):e614–9. Available from: http://www.thelancet.com/article/S2468266721000980/fulltext
24. French MT, Maclean JC, Ettner SL. Drinkers and bettors: Investigating the complementarity of alcohol consumption and problem gambling. Drug Alcohol Depend. 2008 Jul 1;96(1–2):155–64.
25. Kim HS, Hodgins DC, Garcia X, Ritchie E V., Musani I, McGrath DS, et al. A systematic review of addiction substitution in recovery: Clinical lore or empirically-based? Clin Psychol Rev. 2021 Nov 1;89:102083.
26. Kock L, Shahab L, Moore G, Beard E, Bauld L, Reid G, et al. Protocol for expansion of an existing national monthly survey of smoking behaviour and alcohol use in England to Scotland and Wales: The Smoking and Alcohol Toolkit Study. Wellcome Open Res [Internet]. 2021 Mar 29 [cited 2021 Jun 27];6:67. Available from: https://doi.org/10.12688/wellcomeopenres.16700.1
27. Fidler JA, Shahab L, West O, Jarvis MJ, McEwen A, Stapleton JA, et al. "The smoking toolkit study": a national study of smoking and smoking cessation in England. BMC Public Health [Internet]. 2011;11:479. Available from: http://dx.doi.org/10.1186/1471-2458-11-479
28. Ferris JA, Wynne HJ. The Canadian problem gambling index: User manual. Canadian Centre on Substance Abuse; 2001.
29. Holtgraves T. Evaluating the problem gambling severity index. J Gambl Stud [Internet]. 2009 Mar 14 [cited 2023 Jan 12];25(1):105–20. Available from: https://link.springer.com/article/10.1007/s10899-008-9107-7
30. Currie SR, Casey DM, Hodgins DC. Improving the psychometric properties of the Problem Gambling Severity Index. Canadian Consortium for Gambling Research Ottawa; 2010.
31. NRS. National Readership Survey - Social Grade [Internet]. 2017 [cited 2017 Nov 24]. Available from: http://www.nrs.co.uk/nrs-print/lifestyle-and-classification-data/social-grade/
32. Wickham H, Averick M, Bryan J, Chang W, McGowan LD, François R, et al. Welcome to the Tidyverse. J Open Source Softw. 2019;4(43):1686.
33. Lumley T. Analysis of complex survey samples. J Stat Softw. 2004;9:1–19.
34. Roberts A, Sharman S, Coid J, Murphy R, Bowden-Jones H, Cowlishaw S, et al. Gambling and negative life events in a nationally representative sample of UK men. Addictive Behaviors. 2017 Dec 1;75:95–102.
35. Huggett SB, Winiger EA, Corley RP, Hewitt JK, Stallings MC. Alcohol use, psychiatric disorders and gambling behaviors: A multi-sample study testing causal relationships via the co-twin control design. Addictive Behaviors. 2019 Jun 1;93:173–9.
36. Thorne HB, Rockloff MJ, Ferguson SA, Vincent GE, Browne M. Gambling Problems Are Associated with Alcohol Misuse and Insomnia: Results from a Representative National Telephone Survey. International Journal of Environmental Research and Public Health 2021, Vol 18, Page 6683 [Internet]. 2021 Jun 22 [cited 2023 Jun 8];18(13):6683. Available from: https://www.mdpi.com/1660-4601/18/13/6683/htm

For peer review only - http://bmjopen.bmj.com/site/about/guidelines.xhtml

37. Yi S, Kanetkar V. Coping with Guilt and Shame After Gambling Loss. J Gambl Stud [Internet]. 2011 Sep 21 [cited 2023 Jun 9];27(3):371–87. Available from: https://link.springer.com/article/10.1007/s10899-010-9216-y
38. Zack M, Stewart SH, Klein RM, Loba P, Fragopoulos F. Contingent gambling-drinking patterns and problem drinking severity moderate implicit gambling-alcohol associations in problem gamblers. J Gambl Stud [Internet]. 2005 Sep [cited 2023 Jun 9];21(3):325–54. Available from: https://link.springer.com/article/10.1007/s10899-005-3102-z
39. Horn TL, Whelan JP, Weil GT. Does acute alcohol consumption increase risk-taking while gambling? A systematic review and meta-analysis. Addiction. 2022;117(11):2780–90.
40. McGrath DS, Barrett SP. The comorbidity of tobacco smoking and gambling: a review of the literature. Drug Alcohol Rev. 2009;28(6):676–81.
41. Sharman S. The ability of the UK population surveys to capture the true nature of the extent of gambling related harm. Addiction. 2022;
42. Reuter P, Caulkins JP, Midgette G. Heroin use cannot be measured adequately with a general population survey. Addiction. 2021 Oct 1;116(10):2600–9.
43. Sharman S, Dreyer J, Aitken M, Clark L, Bowden-Jones H. Rates of problematic gambling in a British homeless sample: A preliminary study. J Gambl Stud [Internet]. 2015 Jun 1 [cited 2023 Jun 9];31(2):525–32. Available from: https://link.springer.com/article/10.1007/s10899-014-9444-7
44. UK Government. High stakes: gambling reform for the digital age [Internet]. 2023 [cited 2023 Jul 20]. Available from: https://www.gov.uk/government/publications/high-stakes-gambling-reform-for-the-digital-age/high-stakes-gambling-reform-for-the-digital-age

For peer review only

For peer review only - http://bmjopen.bmj.com/site/about/guidelines.xhtml

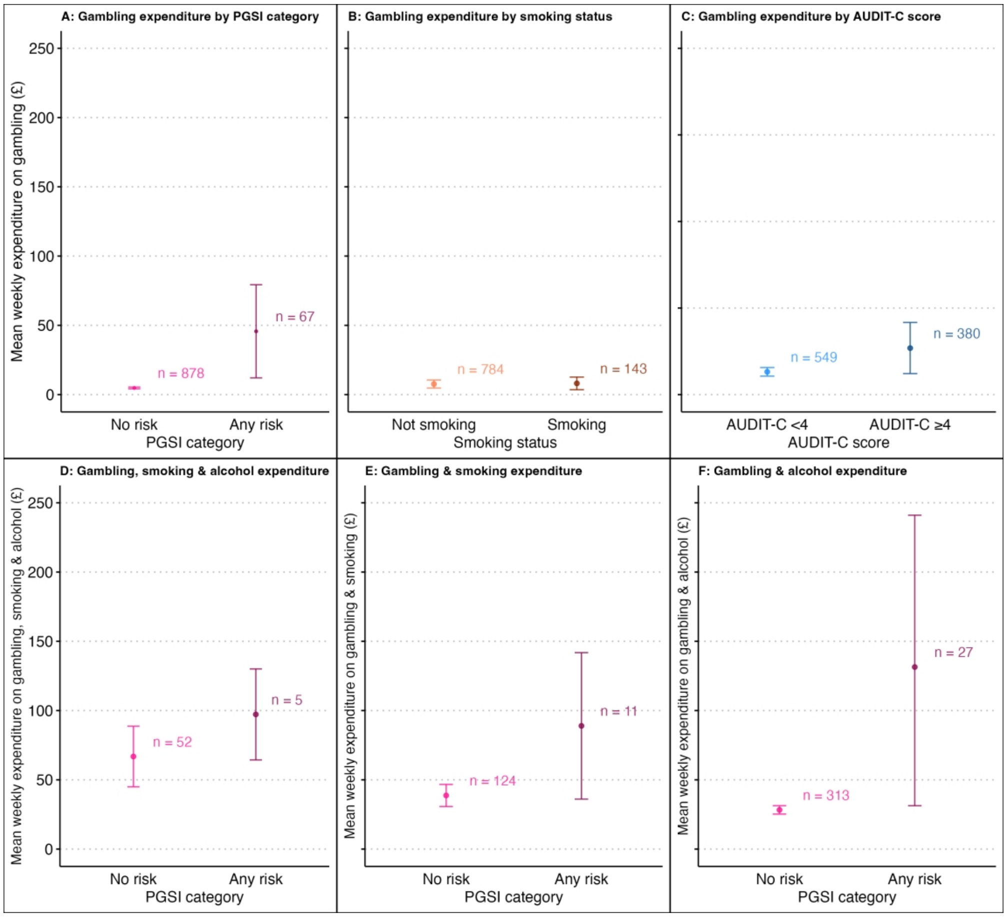

Figure 1: Mean weekly expenditure on gambling according to PGSI category (A), smoking status (B), and AUDIT-C score (C), and mean weekly expenditure on gambling, smoking, and alcohol (D), gambling and smoking (E) and gambling and alcohol (F) according to PGSI category.

710x645mm (118 x 118 DPI)

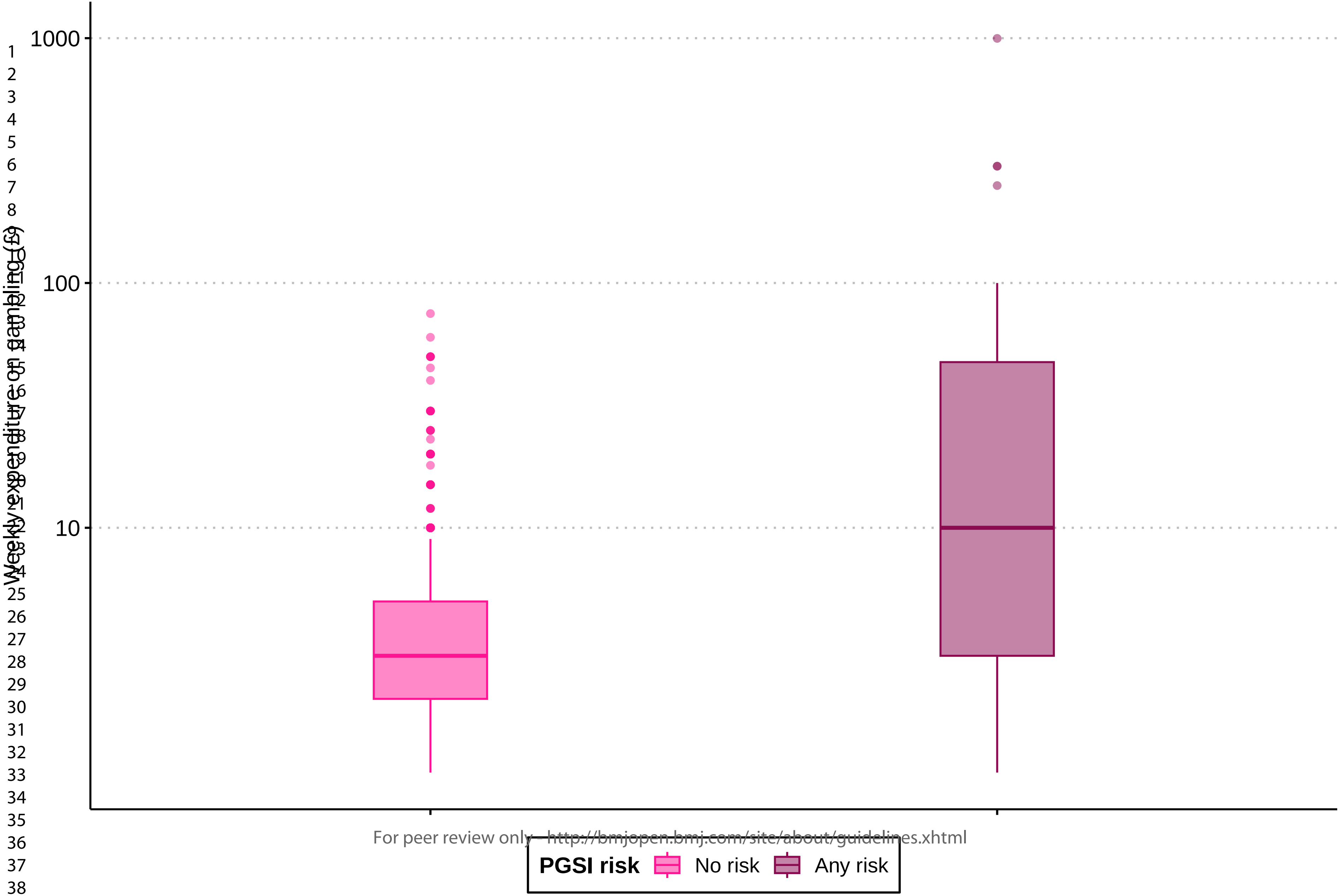
1000
100
10
Weekly expenditure on gambling (£)
PGSI risk
No risk
Any risk

Supplementary appendix

Measures

Past-year gambling

Affirmative responses to any of the gambling types listed below were used to classify individuals as having gambled in the past year.

- National lottery, other lotteries, or scratch cards
- Football pools
- Bingo (not online)
- Slot machines
- Machines in a bookmakers
- Casino table games (not online)
- Online gambling in slots, casino, or bingo
- Online betting with a bookmaker
- Betting exchange
- Horse races (not online)
- Dog races (not online)
- Sports events (not online)
- Private betting
- Loot boxes or skins gambling within online/video games
- Crypto casinos
- Any other gambling event or activity

Problem gambling severity index (PGSI)

The PGSI is a nine item questionnaire on gambling severity and was derived from the Canadian Problem Gambling Index [22,23], and asked to those categorized as having gambled in the past year:

"Thinking about the last 12 months…"

1. Have you bet more than you could really afford to lose?
2. Have you needed to gamble with larger amounts of money to get the same feeling of excitement?
3. When you gambled, did you go back another day to try to win back the money you lost?
4. Have you borrowed money or sold anything to get money to gamble?
5. Have you felt that you might have a problem with gambling?
6. Has gambling caused you any health problems, including stress or anxiety?
7. Have people criticized your betting or told you that you had a gambling problem, regardless of whether or not you thought it was true?
8. Has your gambling caused any financial problems for you or your household?
9. Have you felt guilty about the way you gamble or what happens when you gamble?

For each item the respondent indicates one of the following: Never/None of the time (scored 0); Sometimes (1); Most of the time (2); Almost always (3). A sum score with a possible range from 0 to 27 is calculated. Scores between 1 and 7 represent "at risk" gambling (one to four "low risk" and five to seven "moderate risk"). An individual scoring eight or higher is classified as a

For peer review only - http://bmjopen.bmj.com/site/about/guidelines.xhtml

“disordered gambler” [24]. For this study, a category of “any-risk” will refer to those scoring 1 or greater.

Weekly expenditure on gambling, smoking and alcohol

A sum of weekly expenditure on gambling, smoking and alcohol was derived from responses to three questions on expenditure on each:

“On average about how much per week do you think you spend on [gambling/cigarettes or tobacco/alcohol for your own consumption]?”

Smoking status

Smoking status was ascertained using the following question and response options:

“Which of the following best applies to you?”

1. I smoke cigarettes (including hand rolled) every day
2. I smoke cigarettes (including hand rolled), but not every day
3. I do not smoke cigarettes at all, but I do smoke tobacco of some kind (e.g. Pipe, Cigar or Shisha)
4. I have stopped smoking completely in the last year
5. I stopped smoking completely more than a year ago
6. I have never been a smoker (ie. smoked for a year or more)

Responses of 1, 2 or 4 above were classified as past-year cigarette smokers, 4 or 5 as ex-smokers and 6 as never smokers.

Those who indicated that they do not smoke cigarettes but do smoke tobacco of some kind (answer 3 above) were excluded from the analysis because they did not respond to measures that assess dependence in cigarette smokers (cigarettes per day and time to first cigarette after waking).

Level of alcohol consumption (AUDIT-C score)

Heaviness of the last six months drinking was assessed using the consumption items from the Alcohol Use Disorders Identification Test (AUDIT-C) [26], a three-item screening tool developed by the World Health Organisation, with a score ranging from 0 to 12. Respondents scoring 4 or higher on the AUDIT-C are typically classified as drinking alcohol at increasing and high-risk. For sample characteristics, AUDIT-C was reported as a binary categorical variable (scores less than 4 vs 4 or greater) while for regression modelling it was included as a continuous variable.

Sociodemographic covariates

We used the National Readership Survey’s classification social grade based on occupation (ABC1: higher and intermediate managerial, administrative, and professional, supervisory, clerical and junior managerial, administrative and professional; C2DE: skilled manual workers, semi-skilled and unskilled manual workers and state pensioners, casual and lowest-grade workers, unemployed with state benefits.)[25] .

For peer review only

Sociodemographic covariates included age (18-24, 25-34, 35-44, 45-54 and 65+) identified sex (Women, Men and 'In another way'/refused; all response options were reported in sample characteristics, but due to small case numbers of "in another way/refused" this category was excluded from regression analyses), and region of Great Britain (North, Midlands, South, Scotland and Wales).

Table S1: Types of gambling

Type of gambling	Overall (n=2398)	Smoking (n=345)	Audit-c >4 (n=775)
National lottery, other lotteries of scratch cards	38.4 % (850)	43.8% (138)	45.8% (330)
Football pools	2.1 % (45)	2.3% (8)	4.0% (29)
Online gambling in slots, casino or bingo	4.1% (92)	7.6% (24)	7.9% (57)
Bingo (not online)	3.4% (75)	5.7% (18)	4.8% (35)
Casino table games (not online)	1.7% (39)	2.6% (8)	3.5% (25)
Slot machines	2.8% (62)	6.3% (20)	5.9% (43)
Online betting with bookmaker	5.5% (122)	6.0% (19)	8.0% (58)
Machines in a bookmaker	0.4% (10)	0.0% (0)	0.9% (7)
Horse races (not online)	4.8% (106)	4.9% (16)	7.6% (55)
Dog races (not online)	0.5% (12)	0.3% (1)	0.4% (3)
Sports evens (not online)	1.9% (42)	2.2% (7)	2.8% (20)
Betting exchange	0.4% (9)	0.0% (0)	0.4% (3)
Private betting	0.5% (10)	0.0% (0)	0.6% (4)
Loot boxes or skins gambling within online video games	1.5% (33)	1.9% (6)	1.7% (13)
Crypto casinos	0.0%	0.0% (0)	0.0% (0)
Any other gambling activity	1.6% (36)	1.4% (4)	2.1% (15)

Percentages are weighted

Table S2: Full model output for association between current cigarette smoking, or drinking at increasing and higher risk levels, and any risk gambling according to the PGSI*

**Variable**	**Event Rate**	**OR**	**95% CI**	**P**
**Current cigarette smoking**				
**PGSI category**				
No risk	125 / 897 (14%)	—	—	
Any risk	10 / 57 (18%)	0.80	0.35, 1.66	0.57
**Age**				
18-24	22 / 82 (27%)	—	—	
25-34	36 / 142 (25%)	0.94	0.50, 1.82	0.86
35-44	16 / 146 (11%)	0.36	0.17, 0.76	0.007
45-54	23 / 191 (12%)	0.37	0.18, 0.73	0.004
55-64	24 / 204 (12%)	0.33	0.16, 0.64	0.001
65+	14 / 189 (7%)	0.18	0.08, 0.38	<0.001
Sex				

For peer review only

Men	70 / 499 (14%)	—	—	
Women	65 / 455 (14%)	1.01	0.68, 1.49	0.98
**Region**				
South	44 / 310 (14%)	—	—	
Midlands	24 / 217 (11%)	0.75	0.42, 1.29	0.30
North	36 / 229 (16%)	1.05	0.63, 1.73	0.86
Wales	9 / 69 (13%)	1.09	0.46, 2.36	0.83
Scotland	22 / 129 (17%)	1.20	0.66, 2.16	0.54
**Social grade**				
AB	15 / 241 (6%)	—	—	
C1	54 / 427 (13%)	2.06	1.15, 3.89	0.020
C2	30 / 157 (19%)	3.53	1.82, 7.07	<0.001
D	18 / 69 (26%)	5.15	2.37, 11.3	<0.001
E	18 / 60 (30%)	8.14	3.66, 18.4	<0.001
**Drinking at increasing and higher risk levels**				
**PGSI category**				
No risk	363 / 885 (41%)	—	—	
Any risk	26 / 58 (45%)	0.94	0.52, 1.69	0.83
**Age**				
18-24	48 / 80 (60%)	—	—	
25-34	54 / 140 (39%)	0.42	0.23, 0.76	0.004
35-44	72 / 146 (49%)	0.62	0.35, 1.11	0.11
45-54	75 / 190 (39%)	0.44	0.25, 0.76	0.004
55-64	90 / 202 (45%)	0.54	0.31, 0.94	0.029
65+	50 / 185 (27%)	0.26	0.14, 0.46	<0.001
**Sex**				
Men	247 / 491 (50%)	—	—	
Women	142 / 452 (31%)	0.45	0.34, 0.59	<0.001
**Region**				
South	115 / 304 (38%)	—	—	
Midlands	85 / 213 (40%)	1.14	0.78, 1.67	0.49
North	110 / 235 (47%)	1.63	1.13, 2.35	0.010
Wales	22 / 66 (33%)	0.89	0.49, 1.59	0.70
Scotland	57 / 125 (46%)	1.40	0.89, 2.19	0.14
**Social grade**				
AB	102 / 241 (42%)	—	—	
C1	182 / 421 (43%)	1.04	0.75, 1.46	0.81
C2	73 / 153 (48%)	1.14	0.74, 1.75	0.55
D	27 / 69 (39%)	0.83	0.46, 1.46	0.51

For peer review only

E	5 / 59 (9%)	0.13	0.04, 0.32	<0.001

Table S3: Bayes factors for a range of hypothetical effect sizes related to the association between any risk of harm (according to the PGSI) and smoking status or drinking at increasing and higher risk levels.

Observed OR (95% CI)	Hypothesized OR	Bayes Factor
**PGSI and smoking status**		
	0.50	0.54
	0.90	0.98
0.80 (0.35 - 1.66)	1.10	0.98
	1.50	0.75
	1.90	0.57
**PGSI and AUDIT-C score**		
	0.50	0.40
	0.90	0.95
0.94 (0.52 - 1.69)	1.10	0.95
	1.50	0.60
	1.90	0.43

www.bayesfactor.info

Table S4: Weekly expenditure (in £) on i) gambling according to PGSI, smoking and AUDIT-C scores and ii) expenditure on gambling, smoking and alcohol among people who smoked and/or scored 4 or greater on the AUDIT-C.

	Mean	SD	Lower CI	Upper CI	Median	Min	Max
**Weekly expenditure on gambling**							
**Overall** (n=945)	7.69	37.60	5.17	10.21	3.00	0.00	998.00
**PGSI**							
PGSI No risk (n=878)	4.80	7.68	4.18	5.43	2.00	0.00	75.00
PGSI Any risk (n=67)	45.68	134.13	12.07	79.29	3.00	0.00	998.0
**Smoking status**							
Not smoking (n=784)	7.61	39.67	4.69	10.53	3.00	0.00	998.00
Currently smoking (n=143)	8.09	26.69	3.52	12.65	3.00	0.00	300.00
**AUDIT-C score**							
<4 (n=539)	5.26	10.20	4.27	6.25	2.00	0.00	100.00
4 or higher (n=380)	10.74	56.02	4.86	16.66	3.00	0.00	998.00
**Weekly expenditure on gambling and smoking overall and according to PGSI**							
**Overall** (n=135)	42.73	42.40	33.88	51.59	29.40	2.00	301.00
**PGSI**							
PGSI No Risk (n=124)	38.71	34.48	30.73	46.68	28.00	2.00	170.00
PGSI Any Risk (n=11)	88.94	85.51	36.09	141.80	49.15	18.00	301.00
**Weekly expenditure on gambling and alcohol overall and according to PGSI**							
**Overall** (n=340)	36.48	87.17	26.83	46.13	24.00	1.00	1497.00
**PGSI**							
PGSI No Risk (n=313)	28.29	22.41	25.28	31.30	22.00	1.00	125.00
PGSI Any Risk (n=27)	131.42	288.88	21.81	241.04	50.80	3.00	1497.00
**Weekly expenditure on gambling, smoking and alcohol overall and according to PGSI**							

For peer review only

**Overall** (n=57)	69.37	58.67	48.78	89.96	51.00	3.00	270.00
**PGSI**							
PGSI No Risk (n=52)	66.83	59.75	44.94	88.72	48.92	3.00	270.00
PGSI Any Risk (n=5)	97.22	42.30	64.37	130.06	74.17	30.00	126.00

Figure S1: Box plots for the distribution of mean weekly expenditure (log transformed scale) on gambling according to no risk, or any risk of harm from gambling (scores >0 on the PGSI)

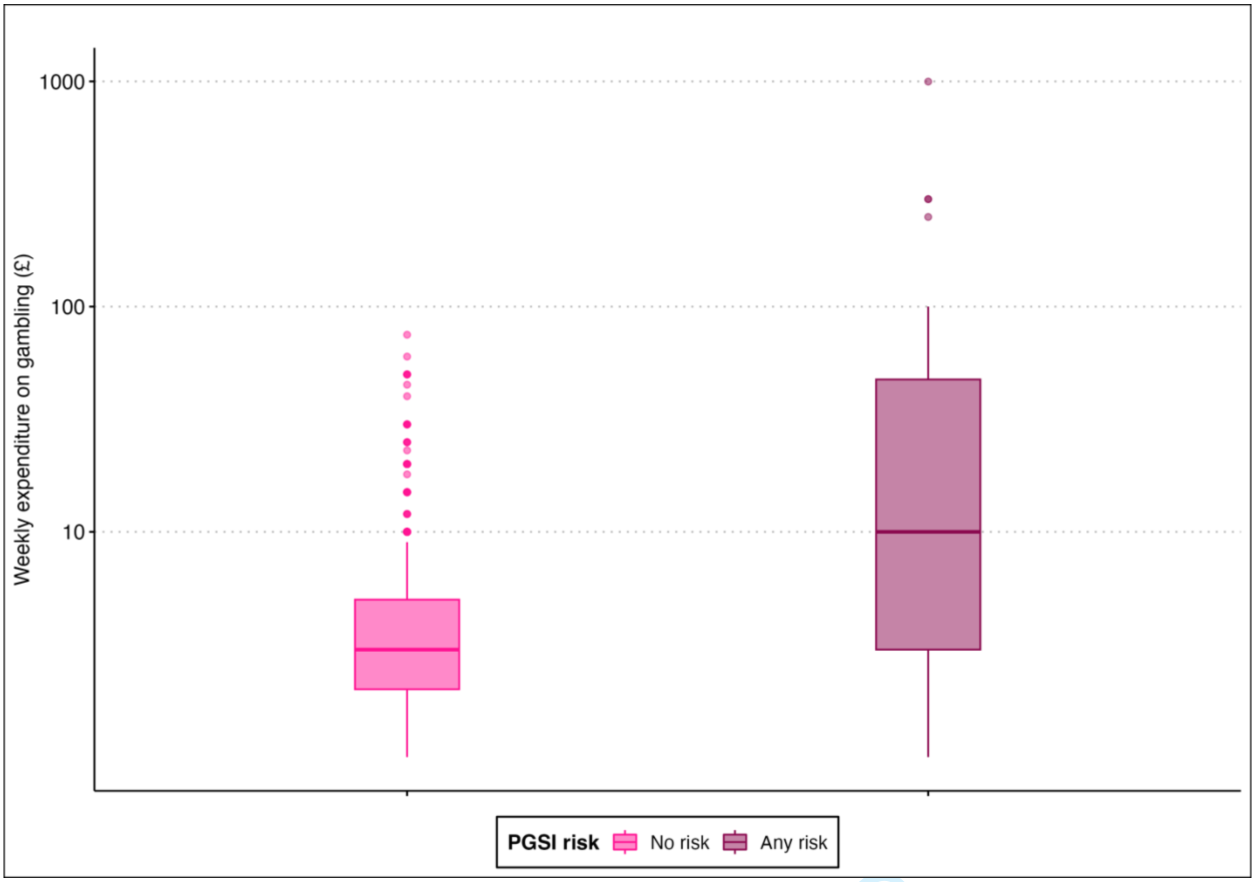

The distribution of mean weekly spend on gambling highlights how the mean is influenced by a small number of higher values in the any risk category:

STROBE 2007 (v4) Statement—Checklist of items that should be included in reports of *cross-sectional studies*

Section/Topic	Item #	Recommendation	Reported on page #
**Title and abstract**	1	(*a*) Indicate the study's design with a commonly used term in the title or the abstract	1
		(*b*) Provide in the abstract an informative and balanced summary of what was done and what was found	1
**Introduction**			
Background/rationale	2	Explain the scientific background and rationale for the investigation being reported	3
Objectives	3	State specific objectives, including any prespecified hypotheses	3
**Methods**			
Study design	4	Present key elements of study design early in the paper	4-6
Setting	5	Describe the setting, locations, and relevant dates, including periods of recruitment, exposure, follow-up, and data collection	4-6
Participants	6	(*a*) Give the eligibility criteria, and the sources and methods of selection of participants	4
Variables	7	Clearly define all outcomes, exposures, predictors, potential confounders, and effect modifiers. Give diagnostic criteria, if applicable	4-6
Data sources/ measurement	8*	For each variable of interest, give sources of data and details of methods of assessment (measurement). Describe comparability of assessment methods if there is more than one group	4-6
Bias	9	Describe any efforts to address potential sources of bias	*4*
Study size	10	Explain how the study size was arrived at	4
Quantitative variables	11	Explain how quantitative variables were handled in the analyses. If applicable, describe which groupings were chosen and why	4-6
Statistical methods	12	(*a*) Describe all statistical methods, including those used to control for confounding	6
		(*b*) Describe any methods used to examine subgroups and interactions	NA
		(*c*) Explain how missing data were addressed	6
		(*d*) If applicable, describe analytical methods taking account of sampling strategy	6
		(*e*) Describe any sensitivity analyses	7
**Results**			

For peer review only

Participants	13*	(a) Report numbers of individuals at each stage of study—eg numbers potentially eligible, examined for eligibility, confirmed eligible, included in the study, completing follow-up, and analysed	7-8
		(b) Give reasons for non-participation at each stage	NA
		(c) Consider use of a flow diagram	NA
Descriptive data	14*	(a) Give characteristics of study participants (eg demographic, clinical, social) and information on exposures and potential confounders	7-8
		(b) Indicate number of participants with missing data for each variable of interest	7-8
Outcome data	15*	Report numbers of outcome events or summary measures	7-8
Main results	16	(*a*) Give unadjusted estimates and, if applicable, confounder-adjusted estimates and their precision (eg, 95% confidence interval). Make clear which confounders were adjusted for and why they were included	7-9
		(*b*) Report category boundaries when continuous variables were categorized	7-10
		(*c*) If relevant, consider translating estimates of relative risk into absolute risk for a meaningful time period	NA
Other analyses	17	Report other analyses done—eg analyses of subgroups and interactions, and sensitivity analyses	7-10
**Discussion**			
Key results	18	Summarise key results with reference to study objectives	10-11
Limitations	19	Discuss limitations of the study, taking into account sources of potential bias or imprecision. Discuss both direction and magnitude of any potential bias	10-11
Interpretation	20	Give a cautious overall interpretation of results considering objectives, limitations, multiplicity of analyses, results from similar studies, and other relevant evidence	10-11
Generalisability	21	Discuss the generalisability (external validity) of the study results	10-11
**Other information**			
Funding	22	Give the source of funding and the role of the funders for the present study and, if applicable, for the original study on which the present article is based	11

*Give information separately for cases and controls in case-control studies and, if applicable, for exposed and unexposed groups in cohort and cross-sectional studies.

Note: An Explanation and Elaboration article discusses each checklist item and gives methodological background and published examples of transparent reporting. The STROBE checklist is best used in conjunction with this article (freely available on the Web sites of PLoS Medicine at http://www.plosmedicine.org/, Annals of Internal Medicine at http://www.annals.org/, and Epidemiology at http://www.epidem.com/). Information on the STROBE Initiative is available at www.strobe-statement.org.

For peer review only
